# Angiotensin II receptor type 1 A1166C modifies the association between angiotensinogen M235T and chronic kidney disease

**DOI:** 10.18632/oncotarget.22121

**Published:** 2017-10-26

**Authors:** Sui-Lung Su, Wei-Teing Chen, Po-Jen Hsiao, Kuo-Cheng Lu, Yuh-Feng Lin, Chin Lin, Wen Su, Shih-Jen Yeh, Hung Chang, Fu-Huang Lin

**Affiliations:** ^1^ School of Public Health, National Defense Medical Center, Taipei, Taiwan; ^2^ Division of Thoracic Medicine, Department of Medicine, Cheng Hsin General Hospital, Taipei, Taiwan; ^3^ Division of Nephrology, Department of Internal Medicine, Tri-Service General Hospital, National Defense Medical Center, Taipei, Taiwan; ^4^ Division of Nephrology, Department of Medicine, Cardinal Tien Hospital, School of Medicine, Fu Jen Catholic University, New Taipei City, Taiwan; ^5^ Division of Nephrology, Department of Medicine, Shuang Ho Hospital, Graduate Institute of Clinical Medicine, Taipei Medical University, Taipei, Taiwan; ^6^ Department of Nursing, Tri-Service General Hospital, Taipei, Taiwan; ^7^ Office of The President, Da-Yeh University, Changhua, Taiwan; ^8^ Department of Physiology and Biophysics, National Defense Medical Center, Taipei, Taiwan

**Keywords:** chronic kidney disease, renin-angiotensin system, polymorphism

## Abstract

Single nucleotide polymorphisms (SNPs) in renin-angiotensin system (RAS) genes are associated with RAS imbalance and chronic kidney disease (CKD). We performed a case-control study and meta-analysis to investigate the association between angiotensinogen (AGT) M235T polymorphism and CKD. A total of 634 patients with end-stage renal disease and 739 healthy controls were studied. We also searched PubMed and the Cochrane Library to identify prospective observational studies published before December 2015. We found that the TT and MT genotypes were associated with a higher risk of CKD than the MM genotype (odds ratio [OR]: 3.56; 95% confidence interval [CI]: 1.14–11.16 and OR: 2.93; 95% CI: 0.91–9.46, respectively). Thirty-eight study populations were included in the meta-analysis. The T allele was associated with a higher risk of CKD than the M allele in all populations (OR: 1.19; 95% CI: 1.08–1.32). The OR was 1.33 in Asians (95% CI: 1.06–1.67) and 1.10 in Caucasians (95% CI: 1.02–1.18). Evaluation of gene-gene and gene-environment interactions using epistasis analysis revealed an interaction between AGT M235T and angiotensin II receptor type 1 A1166C in CKD (OR: 0.767; 95% CI: 0.609–0.965). Genetic testing for CKD in high-risk individuals may be an effective strategy for CKD prevention.

## INTRODUCTION

Renal function gradually decreases in chronic kidney disease (CKD) patients resulting in end-stage renal disease (ESRD). Several studies have reported that the risk of cardiovascular disease mortality was 8–10 times higher among CKD patients compared to other populations, and that the risk increased with decreasing renal function [1]. Risk factors for CKD include diabetes, hyperlipidemia, hypertension, and family history of CKD [2–4]. Diabetes, hyperlipidemia, and hypertension also have strong correlations with heritability [5–7]. Studies have shown that the heritability of serum creatinine was 46% [8]. Thus, genetic factors may have an important role in CKD.

The renin-angiotensin system (RAS) is associated with CKD. If the RAS is overactive, it promotes arterial constriction resulting in an increase in blood pressure and decrease in renal function [9]. The end-product of RAS activity, angiotensin II (Ang II), regulates the synthesis of multiple inflammatory factors associated with CKD such as TNF-alpha, IL-6, MCP-1, and NF-κB [10]. Therefore, single nucleotide polymorphisms (SNPs) in RAS genes may be associated with CKD. Angiotensinogen (AGT) is a component of the RAS. The *AGT* gene is located on chromosome 1 (1q42-43) [11]. It has an overall length of 13 kb and spans five exons and four introns [12]. Excess ATG results in an increase in angiotensin I (Ang I) synthesis, but the total amount of renin remains constant [13]. Ang I is converted into Ang II, which can cause kidney damage. Previous studies have investigated the association between AGT SNPs and CKD. The C803T polymorphism (rs699) has been frequently reported in previous studies. It is located at amino acid 235 in exon 2 and has two possible alleles (M and T). This variant is commonly referred to as M235T [14]. Functional analysis has indicated that individuals with the T allele have higher serum angiotensin concentrations compared to those with the M allele [15]. A meta-analysis verified that individuals with the T allele had a higher risk of hypertension [14] and heart disease [16] compared to those with the M allele. Thus, this locus may be associated with CKD.

Previous meta-analyses have investigated the association between AGT M235T and diabetic nephropathy [14, 17], IgA neuropathy [18], and ESRD [19]. However, these meta-analyses only included data for 9,000 patients. The association between this locus and CKD differed between Caucasian and Asian populations [17–19]. We performed a large-scale case-control study and meta-analysis to investigate the association between AGT M235T and CKD.

## RESULTS

### Case-control study

The basic demographics and blood biochemical tests for the study population are shown in Table 1. The mean age was 64.5 ± 14.9 and 72.7 ± 7.2 years in the case and control groups, respectively. There were 296 men (46.7%) in the case group and 298 (40.2%) in the control group. The mean BMIs were 22.4 ± 4.0 and 24.1 ± 3.2 kg/m^2^ in the case and control groups, respectively. The prevalence of hypertension was 57.8% and 40.8%, and the prevalence of diabetes was 54.2% and 12.3% in the case and control groups, respectively. The mean total cholesterol level was 166.0 ± 36.1 and 191.0 ± 32.5 mg/dL, the mean triglyceride level was 158.5 ± 109.7 and 116.1 ± 60.2 mg/dL and the mean serum creatinine level was 9.6 ± 2.5 and 0.8 ± 0.2 mg/dL in the case and control groups, respectively. Additionally, the mean glomerular filtration rate was 5.5 ± 1.9 mL and 90.5 ± 15.7 mL/min/1.73 m^2^ in the case and control groups, respectively. There were 122 (21.1%) smokers or ex-smokers in the case group and 76 (10.3%) in the control group.

**Table 1:** Characteristics of ESRD and control subjects

	Case (N = 634)	Control (N = 739)	p-value
Age (years)	64.5 ± 14.9	72.7 ± 7.2	< 0.001
Sex (male)	296 (46.7%)	298 (40.2%)	0.015
BMI (kg/m^2^)	22.4 ± 4.0	24.1 ± 3.2	< 0.001
Hypertension	332 (57.8%)	303 (40.8%)	< 0.001
Diabetes mellitus	213 (54.2%)	91 (12.3%)	< 0.001
TC (mg/dL)	166.0 ± 36.1	191.0 ± 32.5	< 0.001
TG (mg/dL)	158.5 ± 109.7	116.1 ± 60.2	< 0.001
Creatinine (mg/dL)	9.6 ± 2.5	0.8 ± 0.2	< 0.001
eGFR (mL/min/1.73m^2^)	5.5 ± 1.9	90.5 ± 15.7	< 0.001
Smoking	122 (21.1%)	76 (10.3%)	< 0.001

TC: Total cholesterol; TG: Triglycerides; eGFR: Estimated glomerular filtration rate.

We calculated the association between the AGT M235T polymorphism and the risk of CKD under genotype, allele frequency, dominant, and recessive models (Table 2). Using the M allele as the reference, the OR for the T allele was 1.27 (95% CI: 1.05–1.55; p = 0.016). The OR was 1.41 after controlling for age, gender, BMI, smoking, hypertension, and diabetes (95% CI: 1.02–1.95; p = 0.035). Similar results were observed with the other genetic models.

**Table 2:** The association between AGT M235T and CKD

	Case	Control	Crude OR(95% CI)	p-value	Adj-OR^a^(95% CI)	p-value	Adj-OR^b^(95% CI)	p-value
Genotype				0.013		0.014		0.066
MM	13 (2.1%)	37 (5.0%)	1		1		1	
MT	168 (26.5%)	205 (27.7%)	2.33 (1.20–4.53)	0.012	2.59 (1.27–5.27)	0.009	2.93 (0.91–9.46)	0.073
TT	453 (71.5%)	497 (67.3%)	2.59 (1.36–4.94)	0.012	2.79 (1.40–5.56)	0.004	3.56 (1.14–11.16)	0.029
Alleles				0.016		0.027		0.035
M-allele	194 (15.3%)	279 (18.9%)	1		1		1	
T-allele	1074 (84.7%)	1199 (81.1%)	1.27 (1.05–1.55)		1.27 (1.03–1.57)		1.41 (1.02–1.95)	
Dominant model				0.093		0.156		0.106
MM	13 (2.1%)	37 (5.0%)	1		1		1	
MT+TT	621 (98.0%)	702 (95.0%)	1.22 (0.97–1.54)		1.20 (0.93–1.54)		1.36 (0.94–1.97)	
Recessive model				0.005		0.004		0.036
MM+MT	181 (28.5%)	242 (32.7%)	1		1		1	
TT	453 (71.5%)	497 (67.3%)	2.52 (1.33–4.78)		3.01 (1.49–6.10)		3.39 (1.09–10.58)	

^a^: Adjustment for age and sex.

^b^: Adjustment for age, sex, BMI, hypertension, diabetes, and smoking status.

### Meta-analysis

**Figure 1:**
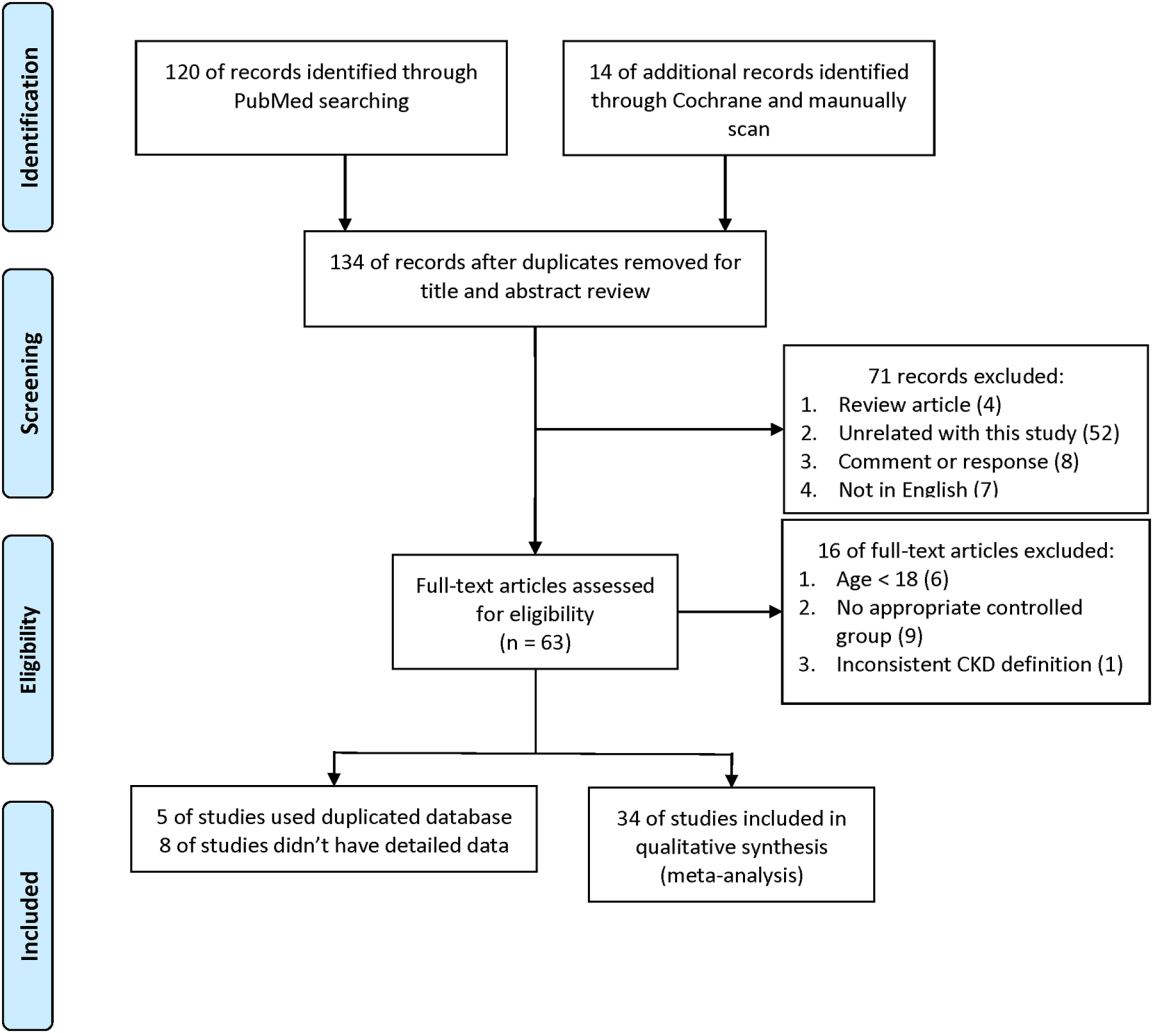
Flow diagram showing the study identification process

The literature review process is shown in Figure 1. We identified 120 publications from PubMed and five from the Cochrane Library (four were also found in PubMed). An additional 13 publications were identified through a search of the reference lists of four published meta-analyses [14, 17–19]. The keywords used in the search and screening processes are shown in Supplementary Table 2. A total of 34 publications were included in our analysis [20–53]. Detailed information for each publication is shown in Supplementary Table 3.

**Figure 2:**
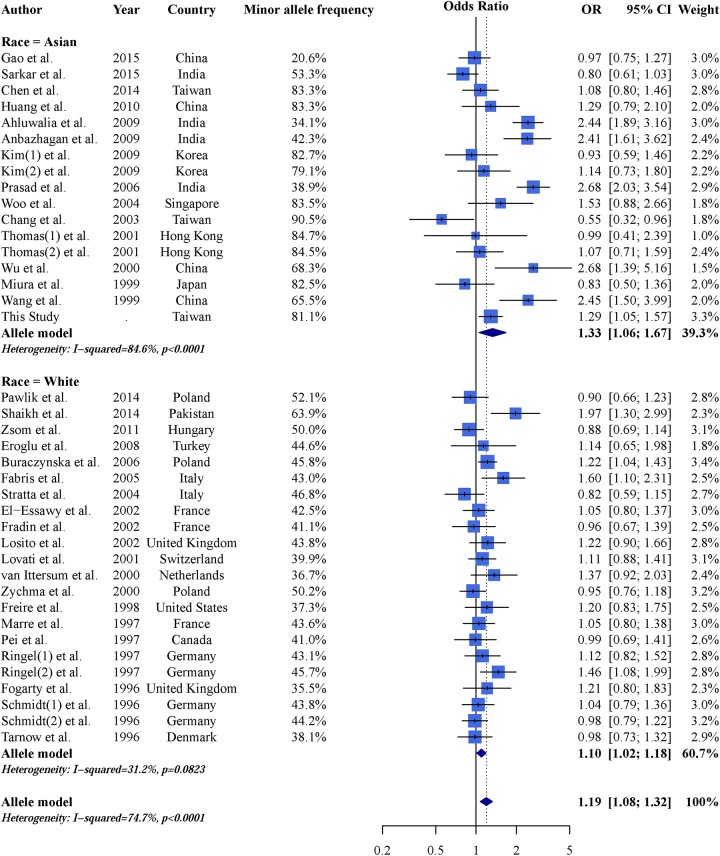
Forest plot of the meta-analysis results demonstrating an association between AGT M235T and CKD under an allele model (reference allele: M)

The results of the meta-analysis under an allele model are shown in Figure 2. We found that the risk ratio of the T allele to the M allele was 1.19 (95% CI: 1.08–1.32). The OR of individuals with the T allele was 1.33 (95% CI: 1.06–1.67) in Asians and 1.10 (95% CI: 1.02–1.18) in Caucasians. The estimated I^2^ was 74.7% for the entire study population, 84.6% for Asians, and 31.2% for Caucasians. Consolidated results for the other genetic models are shown in Table 3. The results were similar between these models (i.e., the risk of CKD increased with the number of T alleles). These results were observed in all populations investigated. However, the Caucasian population did not show significance under a dominant model [OR: 1.08; 95% CI: (0.98–1.18)]. The consolidated results for all studies using the recessive model were asymmetric (Egger's test p = 0.015). Asymmetry could be due to ethnic heterogeneity [54]. We found sufficient symmetry in the analysis of both the Asian and Caucasian subgroups (Egger's test p = 0.064 in Asians; Egger's test p = 0.251 in Caucasians). Therefore, the asymmetry was likely due to ethnic heterogeneity.

**Table 3:** ORs for the association between AGT M235T and CKD under allele type, genotype, dominant, and recessive models

Model	Total				Asian				Caucasian			
	OR	95% CI	I^2^	Egger's test	OR	95% CI	I^2^	Egger's test	OR	95% CI	I^2^	Egger's test
Allele (T vs. M)	1.19	1.08–1.32	74.7%	0.092	1.33	1.06–1.67	84.6%	0.085	1.10	1.02–1.18	31.2%	0.960
Dominant (TT + MT vs. MM)	1.22	1.06–1.40	58.6%	0.129	1.69	1.14–2.49	71.8%	0.061	1.08	0.98–1.18	0.0%,	0.687
Recessive (TT vs. MM + MT)	1.32	1.13–1.53	70.0%	0.015	1.42	1.06–1.89	81.7%	0.064	1.23	1.06–1.42	43.9%	0.251

Egger's test: p value of Egger's regression for asymmetry assessment.

Our data indicated that AGT M235T was associated with CKD. However, we observed high study heterogeneity. Therefore, we investigated whether variables such as race, study design, quality score, kidney function in cases, gender, age, BMI, hypertension, or diabetes mellitus could explain the heterogeneity (Table 4). We used summary information for the case group to analyze gene-environment interactions [55, 56]. However, our data suggested that none of these variables could explain the heterogeneity. Thus, these common environmental factors may not show gene-environment interactions with AGT M235T.

**Table 4:** Effects of moderators on the association between AGT M235T and CKD under an allele model (T vs. M)

	N	τ^2^	Adjust τ^2^	OR	95% CI	p-value^*^	Egger's test p-value
Race	39	0.07443	0.0671	0.832	0.678–1.020	0.0768	0.8492
Study design	39	0.07443	0.07496	0.921	0.731–1.159	0.4817	0.5398
Quality score (per 1 score)	39	0.07443	0.07745	0.961	0.868–1.064	0.4429	0.4812
Kidney function (cases)	39	0.07443	0.07892	0.935	0.727–1.203	0.6015	0.5325
Male gender (per 100%)	36	0.07609	0.07786	0.957	0.509–1.799	0.8915	0.6314
Mean age (per 10 years)	38	0.07606	0.07585	1.074	0.970–1.190	0.1703	0.2877
BMI (per 5 kg/m^2^)	18	0.06223	0.05905	0.830	0.625–1.103	0.1989	0.3689
Hypertension (per 100%)	30	0.05367	0.05148	1.791	0.988–3.248	0.0548	0.3524
Diabetes mellitus (per 100%)	28	0.09401	0.1024	1.028	0.680–1.553	0.8973	0.6313

Dependent variable: log OR of AGT M235T and CKD using allele model.

N: number of studies.

Race: Asian is reference; Study design: cross-sectional study is reference.

^*^: Significance level is p = 0.05/10 (Bonferroni correction).

### Epistasis test in meta-analysis

We next used Epistasis Test in Meta-Analysis (ETMA) to analyze gene-gene interactions [57]. We found that the angiotensin II receptor type 1 (AGTR1) A1166C polymorphism was frequently reported along with the AGT M235T polymorphism. Of the 34 included studies, 18 provided information on AGTR1 A1166C [21, 23, 25–27, 31–35, 37–40, 43, 44, 49, 50]. The results of the ETMA are shown in Table 5. We found that the T allele of AGT M235T [OR: 1.274; 95% CI: (1.174–1.383)] and the C allele of AGTR1 A1166C [OR: 1.296; 95% CI: (1.138–1.476)] were associated with an increased risk of CKD. However, they had a protective effect [OR: 0.767; 95% CI: (0.609–0.965)].

**Table 5:** ETMA of the interaction between AGT M235T and AGTR1 A1166C in CKD

	OR (95% CI)	p-value
AGT M235T (T allele vs. M allele)	1.274 (1.174–1.383)	< 0.001
AGTR1 A1166C (C allele vs. A allele)	1.296 (1.138–1.476)	0.001
AGT M235T × AGTR1 A1166C (interaction term)	0.767 (0.609–0.965)	0.026

## DISCUSSION

We demonstrated a correlation between AGT M235T and CKD. Individuals with the T allele had a higher risk of CKD than those with the M allele. However, we detected high study heterogeneity which confounded the results. Meta-regression analysis indicated that known environmental factors did not modify the correlation between AGT M235T and CKD. However, gene-gene interactions between AGTR1 A1166C and AGT M235T could explain the study heterogeneity. Individuals with the T allele of AGT M235T had higher serum AGT concentrations than those with the M Allele [15]. Excessive ATG can lead to an increase in the concentration of Ang I [13], which is converted into Ang II and can cause kidney damage [10]. Our results are consistent with previous studies that demonstrated an association between RAS and hypertension [14] and heart disease [16].

Several previous meta-analyses have demonstrated a correlation between AGT M235T and CKD [14, 17–19]. However, high study heterogeneity may have impacted the results. We demonstrated gene-gene interactions between AGTR1 A1166C and AGT M235T. Previous studies have found that AGTR1 A1166C may be located in the binding site for microRNA-155 (miR-155), and that the A allele enhances miR-155 binding affinity compared to the C allele resulting in decreased AGTR1 protein expression [58]. Low AGTR1 expression affects Ang II signaling, resulting in decreased synthesis of inflammatory factors such as TNF-alpha, IL-6, MCP-1, and NF-κB [10]. Individuals with the C allele had a higher risk of CKD (OR: 1.296; 95% CI: 1.138–1.476). Similar results were observed in previous studies that investigated the association between AGTR1 A1166C and CKD [59, 60]. Thus, gene-gene interactions exist between AGTR1 A1166C and AGT M235T.

The association between AGT M235T and CKD differed by population. Previous meta-analyses reported a stronger correlation in Asians [14, 17–19]. These results may be explained by the AGTR1 A1166C polymorphism. The 1000 Genomes project reported that the frequency of the C allele was lower in Asians compared to Caucasians [61]. Because the interaction between AGTR1 A1166C and AGT M235T is antagonistic, we would expect an increased risk among Asians compared to Caucasians. Therefore, gene-gene interactions between AGTR1 A1166C and AGT M235T could explain the findings of previous epidemiological studies.

Our study had several limitations. First, we used summary data for the meta-analysis rather than individual patient data. However, previous studies have shown that the inclusion of summary data could increase the sample size and improve the level of evidence [62]. We also performed a case-control study. These results were similar to those of the meta-analysis. Second, we only analysed a few common factors in our gene-environment analysis due to limitations in data availability. Therefore, there may be interactions between AGT M235T and other environmental factors that were not included in our analysis.

We relied on tabular data rather than on individual patient data in our gene-gene interaction analysis, possibly leading to an inflated standard error in pooled analyses. However, we still observed a significant gene-gene interaction between AGT M235T and AGTR1 A1166C in ETMA.

We have demonstrated a correlation between AGT M235T and CKD, which could be modified by AGTR1 A1166C. These data may explain why Asians with the T allele of AGT M235T have a higher risk of CKD. We recommend that patients who are at high-risk for CKD undergo genetic testing.

## MATERIALS AND METHODS

### Case-control study

#### Sample size calculations and study approval

The minimum required sample size was 1,041 subjects. We calculated the size using the following parameters: a two-sided test with a power (1 − β) = 0.8 at a significance level of 0.05, ratio of controls to cases = 1, hypothetical proportion of controls with exposure = 87% and least extreme odds ratio (OR) = 1.5 [63].

We initiated a population-based study at Tri-Service General Hospital (TSGH), a medical teaching hospital of the National Defence Medical Centre in Taipei, Taiwan. The study was approved by the Institutional Ethical Committee of Tri-Service General Hospital (TSGH-1-104-05-006). All subjects enrolled in the study provided written informed consent.

#### Subjects

Subjects in the case group were recruited from dialysis centers in TSGH and Cardinal Tien Hospital. All cases were undergoing dialysis and were diagnosed with ESRD. Control subjects who participated in a check-up program from March 2011 were recruited from the Health Management Centre of TSGH. The inclusion criteria for controls were the following: (1) estimated glomerular filtration rate (eGFR) calculated using the MDRD equation of > 60 mL/min/1.73m^2^, (2) no symptoms of kidney damage such as proteinuria and haematuria, (3) no other diseases such as cancer, and (4) blood sample available for genotyping. Demographic data included age, sex, body mass index (BMI: kg/m^2^), history of hypertension, history of diabetes mellitus, and smoking habits, and were obtained from electronic medical records. Laboratory values including total cholesterol, triglycerides, and creatinine levels, were also collected from medical records. The exclusion criteria for patients were as follows: 1) eGFR of more than 15 mL/min per square meter, 2) diagnosed with cancer. A total of 634 cases (296 men and 338 women) and 739 controls (298 men and 441 women) were included in the study who were treated before July 2015.

#### Genomic DNA extraction and genotyping

Genomic DNA was extracted from peripheral blood samples using standard procedures for proteinase K (Invitrogen, Carlsbad, CA, USA) digestion and phenol/chloroform extraction. Subjects were genotyped using the iPLEX Gold SNP assay to identify AGT M235T polymorphisms [64]. At least 10% of the samples were randomly selected for repeat genotyping to validate the results.

#### Statistical analysis

Continuous variables were evaluated using Student's t tests and reported as the mean ± standard deviation (SD). Genotypes and allelic frequencies were compared between cases and controls using χ^2^ test or Fisher's exact tests. Logistic regression was used to estimate ORs and 95% confidence intervals (CIs) as a measure of the association with CKD susceptibility. The analysis was performed using allele type, co-dominant, dominant, and recessive models. A p < 0.05 was considered significant. Statistical analyses were performed using the R software, version 3.3.1 (R Project for Statistical Computing, Vienna, Austria).

### Meta-analysis

#### Search methods and criteria for study consideration

The PRISMA checklist and Meta-analysis on Genetic Association Studies Checklist is described in Supplementary Table 1 [65]. We compared the risk of CKD between individuals carrying the major (M) and minor (T) alleles of AGT M235T. Relevant studies were identified through a search of PubMed and the Cochrane Library using keywords and medical subject headings that included all spellings of AGT M235T and CKD. The search strategy and records are shown in Supplementary Table 2. We also manually scanned the reference lists of identified trials and review articles to identify additional candidate studies. All articles published prior to December 2015 were eligible for inclusion.

All studies that assessed the association between AGT M235T polymorphisms and CKD risk were considered for inclusion in our analysis. The inclusion criteria were as follows: (1) cross-sectional survey or case-control study, (2) study population age > 18 years, (3) CKD defined according to the National Kidney Foundation: kidney damage by clinical diagnosis or a glomerular filtration rate < 60 mL/min/1.73 m^2^, (4) included at least one control group with normal kidney function, and (5) genotyping data available. Studies that investigated the relationships between genetic polymorphisms and other kidney diseases (e.g. lupus nephritis, polycystic kidney disease, endemic nephropathy, or reflux nephropathy) were excluded from the analysis. If published data was incomplete, we contacted the authors for further information.

#### Data extraction and quality assessment

We recorded the name of the first author, year of publication, ethnicity of the study population, kidney function of cases, case group definitions and characteristics (e.g. mean age, proportion of male subjects, BMI, prevalence of diabetes mellitus, prevalence of hypertension, proportion of smokers, and the AGT M235T genotype distribution). Diabetes mellitus and hypertension were defined by a fasting plasma glucose level of > 126 mg/dL and systolic blood pressure of > 140 mmHg. If the article did not report the prevalence of diabetes mellitus and hypertension or the definitions did not match, we assumed a normal distribution of plasma glucose level and systolic blood pressure.

Risk of bias was assessed using the Newcastle-Ottawa Quality Assessment Scale [66]. The following factors are considered: (1) study population selection, (2) comparability between the case and control groups, and (3) the exposure. Each study received a score between 0 and 9. We investigated the relationship between study quality and the estimation of risk.

### Statistical analysis

The characteristics of the individual study populations are presented as means or proportions where appropriate. We evaluated the association between AGT M235T polymorphisms and CKD risk in each study using ORs and 95% CIs. Heterogeneity was assessed using the τ2 statistic, which was estimated using the DerSimonian-Laird method, and a random-effects model was used to calculate the weighted effect size. Associations between AGT polymorphisms and CKD risk were calculated using an allele type, genotype, dominant, and recessive model.

Egger's regression and funnel plots were used to evaluate the symmetry of the pooled results. The I^2^ was calculated with Cochrane Q tests and used to quantify study heterogeneity. An I^2^ > 50% was indicative of moderate-to-high heterogeneity.

A meta-regression analysis of average summary values was used to explore the source of heterogeneity. According to our previous studies, the average summary value of a case group can be used to build a model and can facilitate interaction effect estimation [29]. An interaction effect is determined using the OR and defined as the ratio between ORs per 1 unit. Possible moderators (race, study design, quality score, kidney function of case, sex, age, BMI, hypertension, diabetes mellitus and smoking) were tested to explore heterogeneity.

For further explain the unexplained heterogeneity between included studies, we considered explore gene-gene interaction in our meta-analysis. Although meta-regression is a common approach to assessing interaction effects in meta-analysis, but it is not suitable for analyzing gene-gene interaction. The most important problem is attenuation bias, and these random errors will lead to inconsistent estimates of interaction effects but this phenomenon does not occur in individual data analysis [57]. ETMA (Epistasis Test in Meta-Analysis) is a Markov Chain Monte Carlo based method for consistency the estimate. We used the “etma” package of R software to implement this analysis.

This study considered a p value of <0.05 to be significant. However, because of multiple comparison correction, a p < 0.05 was considered significant. Statistical analyses were conducted using the ‘metafor’ and ‘meta’ packages for the R software, version 3.3.1.

## SUPPLEMENTARY MATERIALS FIGURES AND TABLES








