# Supplementary material for: Angiotensin II receptor type 1 A1166C modifies the association between angiotensinogen M235T and chronic kidney disease
Source: Oncotarget. 2017 Oct 26;8(64):107833–43. doi: 10.18632/oncotarget.22121 (PMC5746107; doi:10.18632/oncotarget.22121)
Supplement: Supplementary file 1 [file oncotarget-08-107833-s001.pdf]

Angiotensin II receptor type 1 A1166C modifies the association between angiotensinogen M235T and chronic kidney disease

SUPPLEMENTARY MATERIALS

Supplementary Table 1: PRISMA 2009 Checklist

See Supplementary File 1

Supplementary Table 2: Search strategies and detailed records

See Supplementary File 2

Supplementary Table 3: Summary of studies included in the meta-analysis

See Supplementary File 3
