# Supplementary material for: Angiotensin II receptor type 1 A1166C modifies the association between angiotensinogen M235T and chronic kidney disease
Source: Oncotarget. 2017 Oct 26;8(64):107833–43. doi: 10.18632/oncotarget.22121 (PMC5746107; doi:10.18632/oncotarget.22121)
Supplement: Supplementary file 3 [file oncotarget-08-107833-s003.docx]

**Supplementary Table 2: Search strategies and detailed records**

| **Relevant text of AGT M235T**   1. Angiotensinogen 2. Hypertensinogen 3. AGT 4. M235T 5. rs699 6. ((1 or 2 or 3)and 4) or 5 | **Relevant text of chronic kidney disease**   1. End-Stage Kidney Disease 2. End-Stage Renal Disease 3. End-Stage Renal Failure 4. ESRD 5. ESRF 6. ESKD 7. Chronic kidney disease 8. CKD 9. dialysis 10. eGFR 11. estimated Glomerular filtration rate 12. nephropathy 13. 7 or 8 or 9 or 10 or 11 or 12 or 13 or 14 or 15 or 16 or 17 or 18   **Combined (Final strategy)**  6 and 19 |
| --- | --- |

**Web sites and uniform resource locator:**PubMed: <http://www.ncbi.nlm.nih.gov/pubmed>
Cochrane Library: <http://www.thecochranelibrary.com>

**Records from PUBMED [1-120]:**Unrelated records (wrong outcome) [1-85]
Duplicated samples [86, 87]
Paper didn’t provide detailed genotyping data [88-92]
Included studies [93-120]

**Records from Cochrane Library:**Duplicated records [29, 38, 71]

**Records from manually scan:**

Unrelated records (wrong outcome) [121-125]

Duplicated samples [126-128]

Paper didn’t provide detailed genotyping data [129]

Included studies [130-133]

**REFERENCES**

1. Zhou TB, Yin SS, Qin YH. Association of angiotensinogen M235T gene polymorphism with end-stage renal disease risk: a meta-analysis. Mol Biol Rep. 2013; 40:765-72. doi: 10.1007/s11033-012-2114-x.
2. Ding W, Wang F, Fang Q, Zhang M, Chen J, Gu Y. Association between two genetic polymorphisms of the renin-angiotensin-aldosterone system and diabetic nephropathy: a meta-analysis. Mol Biol Rep. 2012; 39:1293-303. doi: 10.1007/s11033-011-0862-7.
3. Staessen JA, Kuznetsova T, Wang JG, Emelianov D, Vlietinck R, Fagard R. M235T angiotensinogen gene polymorphism and cardiovascular renal risk. J Hypertens. 1999; 17:9-17.
4. Woo KT, Lau YK, Choong HL, Tan HK, Foo MW, Lee EJ, Anantharaman V, Lee GS, Yap HK, Yi Z, Fook-Chong S, Wong KS, Chan CM. Genomics and disease progression in IgA nephritis. Annals of the Academy of Medicine, Singapore. 2013; 42:674-80.
5. Alves M, Souza e Silva NA, Salis LH, Pereira Bde B, Godoy PH, Nascimento EM, Oliveira JM. Survival and predictive factors of lethality in hemodialysis: D/I polymorphism of the angiotensin I-converting enzyme and of the angiotensinogen M235T genes. Arquivos brasileiros de cardiologia. 2014; 103:209-19.
6. Mao S, Huang S. Association of angiotensinogen gene M235T polymorphism with the risk of IgA nephropathy: a meta-analysis. Renal failure. 2014; 36:466-72. doi: 10.3109/0886022x.2013.868318.
7. Mao S, Huang S. Association of AGT M235T gene polymorphism with HSP/HSPN risk. Renal failure. 2015; 37:16-21. doi: 10.3109/0886022x.2014.977142.
8. Tang W, Zhou TB, Jiang Z. RETRACTED: Association of the angiotensinogen M235T gene polymorphism with risk of diabetes mellitus developing into diabetic nephropathy. J Renin Angiotensin Aldosterone Syst. 2015; 16:Np27-34. doi: 10.1177/1470320314563426.
9. Kaplan I, Sancaktar E, Ece A, Sen V, Tekkesin N, Basarali MK, Kelekci S, Evliyaoglu O. Gene polymorphisms of adducin GLY460TRP, ACE I/D, AND AGT M235T in pediatric hypertension patients. Medical science monitor. 2014; 20:1745-50. doi: 10.12659/msm.892140.
10. Yang CH, Zhou TB. RETRACTED: Relationship between the angiotensinogen A1166C gene polymorphism and the risk of diabetes mellitus developing into diabetic nephropathy. J Renin Angiotensin Aldosterone Syst. 2015; 16:Np45. doi: 10.1177/1470320314566221.
11. Chudek J, Szotowska M, Karkoszka H, Verbeke F, Trautsolt W, Gumprecht J, Vanholder R, Wiecek A. Genotypes of renin-angiotensin system and plasma adiponectin concentration in kidney transplant patients. Annals of transplantation. 2013; 18:593-603. doi: 10.12659/aot.884022.
12. Wang AY, Chan JC, Wang M, Poon E, Lui SF, Li PK, Sanderson J. Cardiac hypertrophy and remodeling in relation to ACE and angiotensinogen genes genotypes in Chinese dialysis patients. Kidney Int. 2003; 63:1899-907. doi: 10.1046/j.1523-1755.2003.00933.x.
13. Ozdemir BH, Ozdemir FN, Atac FB, Ozdemir AA, Haberal M. Angiotensinogen t235 and angiotensin-converting enzyme insertion/deletion polymorphisms associated with the development of posttransplantation diabetes mellitus in renal allograft recipients. Transplantation proceedings. 2011; 43:572-4. doi: 10.1016/j.transproceed.2011.01.046.
14. Yugar-Toledo JC, Martin JF, Krieger JE, Pereira AC, Demacq C, Coelho OR, Pimenta E, Calhoun DA, Junior HM. Gene variation in resistant hypertension: multilocus analysis of the angiotensin 1-converting enzyme, angiotensinogen, and endothelial nitric oxide synthase genes. DNA and cell biology. 2011; 30:555-64. doi: 10.1089/dna.2010.1156.
15. Raizada V, Skipper B, Luo W, Garza L, Hines CW, Harford AA, Zager PG, Griffith J, Raj D, Spalding CT. Renin-angiotensin polymorphisms and QTc interval prolongation in end-stage renal disease. Kidney Int. 2005; 68:1186-9. doi: 10.1111/j.1523-1755.2005.00510.x.
16. Zhou TB, Li HY, Jiang ZP, Zhou JF, Huang MF, Zhou ZY. RETRACTED: Role of renin-angiotensin-aldosterone system inhibitors in radiation nephropathy. J Renin Angiotensin Aldosterone Syst. 2015; 16:Np11. doi: 10.1177/1470320314563424.
17. Teranishi J, Yamamoto R, Nagasawa Y, Shoji T, Iwatani H, Okada N, Moriyama T, Yamauchi A, Tsubakihara Y, Imai E, Rakugi H, Isaka Y. ACE insertion/deletion polymorphism (rs1799752) modifies the renoprotective effect of renin-angiotensin system blockade in patients with IgA nephropathy. J Renin Angiotensin Aldosterone Syst. 2015; 16:633-41. doi: 10.1177/1470320313515036.
18. Prasad GV, Pinnaduwage D, Parkes RK, Midgley J, Balfe JW, Hegele RA, Bull SB, Cole EH, Logan AG. Angiotensinogen M235T genotype predicts progression in chronic renal allograft dysfunction. Transplantation. 2003; 75:209-16. doi:10.1097/01.tp.0000040866.14123.05.
19. Baboolal K, Ravine D, Daniels J, Williams N, Holmans P, Coles GA, Williams JD. Association of the angiotensin I converting enzyme gene deletion polymorphism with early onset of ESRF in PKD1 adult polycystic kidney disease. Kidney Int. 1997; 52:607-13.
20. Saggar-Malik AK, Afzal AR, Swissman JS, Bland M, Sagnella GA, Eastwood JB, MacGregor GA, Jeffery S. Lack of association of ACE/angiotensinogen genotype with renal function in autosomal dominant polycystic kidney disease. Genetic testing. 2000; 4:299-303. doi: 10.1089/10906570050501542.
21. Bettinaglio P, Galbusera A, Caprioli J, Orisio S, Perna A, Arnoldi F, Bucchioni S, Noris M. Single Strand Conformation Polymorphism (SSCP) as a quick and reliable method to genotype M235T polymorphism of angiotensinogen gene. Clinical biochemistry. 2002; 35:363-8.
22. Narita I, Goto S, Saito N, Song J, Omori K, Kondo D, Sakatsume M, Gejyo F. Angiotensinogen gene variation and renoprotective efficacy of renin-angiotensin system blockade in IgA nephropathy. Kidney Int. 2003; 64:1050-8. doi:10.1046/j.1523-1755.2003.00187.x.
23. Kujawa-Szewieczek A, Kolonko A, Kocierz M, Szotowska M, Trusolt W, Karkoszka H, Gumprecht J, Chudek J, Wiecek A. Association between gene polymorphisms of the components of the renin-angiotensin-aldosteron system, graft function, and the prevalence of hypertension, anemia, and erythrocytosis after kidney transplantation. Transplantation proceedings. 2011; 43:2957-63. doi: 10.1016/j.transproceed.2011.07.016.
24. Reis K, Arinsoy T, Derici U, Gonen S, Bicik Z, Soylemezoglu O, Yasavul U, Hasanoglu E, SindelS. Angiotensinogen and plasminogen activator inhibitor-1 gene polymorphism in relation to chronic allograft dysfunction. Clinical transplantation. 2005; 19:10-4. doi: 10.1111/j.1399-0012.2004.00187.x.
25. Boger CA, Gotz AK, Kruger B, Hosl M, Schmitz G, Riegger GA, Kramer BK. Effect of genetic variation on therapy with angiotensin converting enzyme inhibitors or angiotensin receptor blockers in dialysis patients. European journal of medical research. 2005; 10:161-8.
26. Goto S, Narita I, Saito N, Watanabe Y, Yamazaki H, Sakatsume M, Shimada, H, Nishi S, Ueno M, Akazawa K, Arakawa M, Gejyo F. A(-20)C polymorphism of the angiotensinogen gene and progression of IgA nephropathy. Kidney Int. 2002; 62:980-5. doi: 10.1046/j.1523-1755.2002.00517.x.
27. Yoshida H, Kuriyama S, Atsumi Y, Tomonari H, Mitarai T, Hamaguchi A, Kubo H, Kawaguchi Y, Kon V, Matsuoka K, Ichikawa I, Sakai O. Angiotensin I converting enzyme gene polymorphism in non-insulin dependent diabetes mellitus. Kidney Int. 1996; 50:657-64.
28. Jacobsen P, Tarnow L, Carstensen B, Hovind P, Poirier O, Parving HH. Genetic variation in the Renin-Angiotensin system and progression of diabetic nephropathy. J Am Soc Nephrol. 2003; 14:2843-50.
29. Lee YJ, Jang HR, Kim SG, Chae DW, Do JY, Lee JE, Huh W, Kim DJ, Oh HY, Kim YG. Renoprotective efficacy of valsartan in chronic non-diabetic proteinuric nephropathies with renin-angiotensin system gene polymorphisms. Nephrology (Carlton). 2011; 16:502-10. doi: 10.1111/j.1440-1797.2011.01448.x.
30. Coll E, Campos B, Gonzalez-Nunez D, Botey A, Poch E. Association between the A1166C polymorphism of the angiotensin II receptor type 1 and progression of chronic renal insufficiency. Journal of nephrology. 2003; 16:357-64.
31. Retraction notice. J Renin Angiotensin Aldosterone Syst. 2015; 16:Np10. Edoi: 10.1177/1470320315623881.
32. Zhong W, Jiang Z, Zhou TB. RETRACTED: Association between the ACE I/D gene polymorphism and T2DN susceptibility: The risk of T2DM developing into T2DN in the Asian population. J Renin Angiotensin Aldosterone Syst. 2015; 16:Np35. doi: 10.1177/1470320314566019.
33. Yang CH, Zhou TB. RETRACTED: Association of the ACE I/D gene polymorphism with sepsis susceptibility and sepsis progression. J Renin Angiotensin Aldosterone Syst. 2015; 16:Np51. doi: 10.1177/1470320314568521.
34. Konoshita T, Miyagi K, Onoe T, Katano K, Mutoh H, Nomura H, Koni I, Miyamori I, Mabuchi H. Effect of ACE gene polymorphism on age at renal death in polycystic kidney disease in Japan. Am J Kidney Dis. 2001; 37:113-8.
35. Chapdelaine I, Goupil R, Azcoitia V, Rioux JP, Raymond-Carrier S, Madore F, Troyanov S. Gene polymorphisms as clinical tools in chronic glomerulopathies: a prospective study. Nephron Clinical practice. 2012; 121:c174-9. doi: 10.1159/000346404.
36. Siekierka-Harreis M, Kuhr N, Willers R, Ivens K, Grabensee B, Mondry A, Loh MC, Rump LC, Blume C. Impact of genetic polymorphisms of the renin-angiotensin system and of non-genetic factors on kidney transplant function--a single-center experience. Clinical transplantation. 2009; 23:606-15. doi: 10.1111/j.1399-0012.2009.01033.x.
37. Goyache-Goni B, Aranda-Lara P, Reyes-Engels A, Frutos-Sanz MA, Hernandez-Marrero D. The influence of renin-angiotensin system genotypes on the antiproteinuric response to high doses of olmesartan in non-diabetic proteinuric nephropathies. Nefrologia. 2013; 33:771-8. doi: 10.3265/Nefrologia.pre2013.Sep.12258.
38. Amara AB, Sharma A, Alexander JL, Alfirevic A, Mohiuddin A, Pirmohamed M, Close GL, Grime S, Maltby P, Shawki H, Heyworth S. Randomized controlled trial: lisinopril reduces proteinuria, ammonia, and renal polypeptide tubular catabolism in patients with chronic allograft nephropathy. Transplantation. 2010; 89:104-14. doi: 10.1097/TP.0b013e3181bf13d9.
39. Procopciuc LM, Sitar-Taut A, Pop D, Sitar-Taut DA, Olteanu I, Zdrenghea D. Renin angiotensin system polymorphisms in patients with metabolic syndrome (MetS). European journal of internal medicine. 2010; 21:414-8. doi: 10.1016/j.ejim.2010.06.001.
40. Reis KA, Onal B, Gonen S, Arinsoy T, Erten Y, Ilgit E, Soylemezoglu O, Derici U, Guz G, Bali M, Sindel S. Angiotensinogen and plasminogen activator inhibitor-1 gene polymorphism in relation to renovascular disease. Cardiovascular and interventional radiology. 2006; 29:59-63. doi: 10.1007/s00270-005-0072-6.
41. Kuriyama S, Tomonari H, Tokudome G, Kaguchi Y, Hayashi H, Kobayashi H, Horiguchi M, Ishikawa M, Hara Y, Hosoya T. Association of angiotensinogen gene polymorphism with erythropoietin-induced hypertension: a preliminary report. Hypertension research. 2001; 24:501-5.
42. Tarnow L, Kjeld T, Knudsen E, Major-Pedersen A, Parving HH. Lack of synergism between long-term poor glycaemic control and three gene polymorphisms of the renin angiotensin system on risk of developing diabetic nephropathy in type I diabetic patients. Diabetologia. 2000; 43:794-9. doi: 10.1007/s001250051377.
43. Ozkaya O, Soylemezoglu O, Gonen S, Misirlioglu M, Tuncer S, Kalman S, Buyan N, Hasanoglu E. Renin-angiotensin system gene polymorphisms: association with susceptibility to Henoch-Schonlein purpura and renal involvement. Clinical rheumatology. 2006; 25:861-5. doi: 10.1007/s10067-006-0207-4.
44. Akcay A, Micozkadioglu H, Atac FB, Agca E, Ozdemir FN. Relationship of ENOS and RAS gene polymorphisms to initial peritoneal transport status in peritoneal dialysis patients. Nephron Clinical practice. 2006; 104:c41-6. doi: 10.1159/000093669.
45. Akcay A, Sezer S, Ozdemir FN, Arat Z, Atac FB, Verdi H, Colak T, Haberal M. Association of the genetic polymorphisms of the renin-angiotensin system and endothelial nitric oxide synthase with chronic renal transplant dysfunction. Transplantation. 2004; 78:892-8.
46. Wong TY, Szeto CC, Chow KM, Chan JC, Li PK. Contribution of gene polymorphisms in the renin-angiotensin system to macroangiopathy in patients with diabetic nephropathy. Am J Kidney Dis. 2001; 38:9-17. doi: 10.1053/ajkd.2001.25175.
47. Zhang G, Wang H, Wang F, Yu L, Yang X, Meng J, Qin W, Wu G, Li J, Yang A, Zhou Y. Gene polymorphisms of the renin-angiotensin-aldosterone system and angiotensin II type 1-receptor activating antibodies in renal rejection. Tohoku J Exp Med. 2007; 213:203-14.
48. Thameem F, Voruganti VS, He X, Nath SD, Blangero J, MacCluer JW, Comuzzie AG, Abboud HE, Arar NH. Genetic variants in the renin-angiotensin system genes are associated with cardiovascular-renal-related risk factors in Mexican Americans. Human genetics. 2008; 124:557-9. doi: 10.1007/s00439-008-0581-x.
49. Manea SA, Robciuc A, Guja C, Heltianu C. Identification of gene variants in NOS3, ET-1 and RAS that confer risk and protection against microangiopathy in type 2 diabetic obese subjects. Biochemical and biophysical research communications. 2011; 407:486-90. doi: 10.1016/j.bbrc.2011.03.043.
50. Ito H, Tsukui S, Kanda T, Utsugi T, Ohno T, Kurabayashi M. Angiotensin-converting enzyme insertion/deletion polymorphism and polyneuropathy in type 2 diabetes without macroalbuminuria. The Journal of international medical research. 2002; 30:476-82.
51. Sprovieri SR, Sens YA, Martini Filho D. Association between polymorphisms of the renin-angiotensin system and more severe histological forms of lupus nephritis. Clinical nephrology. 2005; 64:20-7.
52. Akcay A, Ozdemir FN, Atac FB, Sezer S, Verdi H, Arat Z, Atac FB, Verdi H, Colak T, Haberal M. Angiotensin-converting enzyme genotype is a predictive factor in the peak panel-reactive antibody response. Transplantation proceedings. 2004; 36:35-7. doi: 10.1016/j.transproceed.2003.11.013.
53. Olivieri O, Trabetti E, Grazioli S, Stranieri C, Friso S, Girelli D, Russo C, Pignatti PF, Mansueto G, Corrocher R. Genetic polymorphisms of the renin-angiotensin system and atheromatous renal artery stenosis. Hypertension. 1999; 34:1097-100.
54. Ozen S, Alikasifoglu M, Saatci U, Bakkaloglu A, Besbas N, Kara N, Kocak H, Erbas B, Umsal I, Tuncbilek E. Implications of certain genetic polymorphisms in scarring in vesicoureteric reflux: importance of ACE polymorphism. Am J Kidney Dis. 1999; 34:140-5. doi: 10.1053/ajkd03400140.
55. Nicod J, Richard A, Frey FJ, Ferrari P. Recipient RAS gene variants and renal allograft function. Transplantation. 2002; 73:960-5.
56. Argani H, Noroozianavval M, Aghaeishahsavari M, Veisi P, Rashtchizadeh N, Ghorbanihaghjo A, Bonyadi M, Asgarzadeh M, Hamzeiy H. Renin-angiotensin system polymorphisms and renal graft function in renal transplant recipients. Saudi medical journal. 2007; 28:1496-502.
57. Slowinski T, Diehr P, Kleemann P, Fritsche L, Renders L, Budde K, Hauser IA, Neumayer HH, Hocher B. No association between renin-angiotensin system gene polymorphisms and early and long-term allograft dysfunction in kidney transplant recipients. Nephrol Dial Transplant. 2004; 19:2846-51. doi: 10.1093/ndt/gfh483.
58. Pontremoli R, Ravera M, Viazzi F, Nicolella C, Berruti V, Leoncini G, Comuzzie AG, Abbounf HE, Arar NH. Genetic polymorphism of the renin-angiotensin system and organ damage in essential hypertension. Kidney Int. 2000; 57:561-9. doi: 10.1046/j.1523-1755.2000.00876.x.
59. Bofinger A, Hawley C, Fisher P, Daunt N, Stowasser M, Gordon R. Polymorphisms of the renin-angiotensin system in patients with multifocal renal arterial fibromuscular dysplasia. Journal of human hypertension. 2001; 15:185-90. doi: 10.1038/sj.jhh.1001144.
60. Forrest EH, Thorburn D, Spence E, Oien KA, Inglis G, Smith CA,McCruden EA, FOX R, Mills PR. Polymorphisms of the renin-angiotensin system and the severity of fibrosis in chronic hepatitis C virus infection. Journal of viral hepatitis. 2005; 12:519-24. doi: 10.1111/j.1365-2893.2005.00630.x.
61. Parsa A, Lovett DH, Peden EA, Zhu L, Seldin MF, Criswell LA. Renin-angiotensin system gene polymorphisms predict the progression to renal insufficiency among Asians with lupus nephritis. Genes and immunity. 2005; 6:217-24. doi: 10.1038/sj.gene.6364179.
62. Conen D, Glynn RJ, Buring JE, Ridker PM, Zee RY. Association of renin-angiotensin and endothelial nitric oxide synthase gene polymorphisms with blood pressure progression and incident hypertension: prospective cohort study. J Hypertens. 2008; 26:1780-6. doi: 10.1097/HJH.0b013e3283077eef.
63. Kujawa-Szewieczek A, Kocierz M, Piecha G, Kolonko A, Chudek J, Wiecek A. [Gene polymorphisms of renin-angiotensin-aldosterone system components and the progression of chronic kidney diseases]. [Article in Polish]. Postepy higieny i medycyny doswiadczalnej. 2010; 64:423-38.
64. Wang J, Zhu X, Yang L, Zhou W, Li H, Fu H, Zhu L, Yuan S. [Relationships of angiotensinogen gene M235T variant with diabetic nephropathy in Chinese type 2 diabetes mellitus]. [Article in Chinese]. Zhonghua yi xue yi chuan xue za zhi. 1999; 16:299-302.
65. Buraczynska M, Jozwiak L, Spasiewicz D, Nowicka T, Ksiazek A. [Renin-angiotensin system genes in chronic glomerulonephritis]. Polskie Archiwum Medycyny Wewnetrznej. 2001; 105:455-60.
66. Buraczynska M, Ksiazek P, Lopatynski J, Spasiewicz D, Nowicka T, Ksiazek A. [Association of the renin-angiotensin system gene polymorphism with nephropathy in type II diabetes]. [Article in Polish]. Polskie Archiwum Medycyny Wewnetrznej. 2002; 108:725-30.
67. Coto E, Marin R, Alvarez V, Praga M, Fernandez Andrade C, Arias M, Poveda R, Valles M, Galceran JM, Luno J, Rivena F, Campistol JM. [Pharmacogenetics of angiotensin system in non diabetic nephropathy]. [Article in Spanish]. Nefrologia. 2005; 25:381-6.
68. Bzoma B, Debska-Slizien A, Dudziak M, Raczynska K, Slizien W, Brylowska A, Rutkowski B. [Genetic predisposition to systemic complications of arterial hypertension in maintenance haemodialysis patients]. [Article in Polish]. Polski merkuriusz lekarski. 2008; 25:209-16.
69. Jeunemaitre X. [Genetic polymorphisms in the renin-angiotensin system]. [Article in French]. Therapie. 1998; 53:271-7.
70. Buraczynska M, Grzebalska A, Spasiewicz D, Orlowska G, Ksiazek A. Genetic polymorphisms of renin-angiotensin system and progression of interstitial nephritis. Annales Universitatis Mariae Curie-Sklodowska Sectio D: Medicina. 2002; 57:330-6.
71. Ong-Ajyooth S, Ong-Ajyooth L, Limmongkon A, Tiensingh A, Parichatikanon P, Nilwarangkur S. The renin--angiotensin system gene polymorphisms and clinicopathological correlations in IgA nephropathy. Journal of the Medical Association of Thailand. 1999; 82:681-9.
72. Gallego PH, Shephard N, Bulsara MK, van Bockxmeer FM, Powell BL, Beilby JP, Arscott G, Le Page M, Palmer LJ, Davis EA, Jones TW, Choong CS . Angiotensinogen gene T235 variant: a marker for the development of persistent microalbuminuria in children and adolescents with type 1 diabetes mellitus. J Diabetes Complications. 2008; 22:191-8. doi: 10.1016/j.jdiacomp.2007.03.003.
73. Maruyama K, Yoshida M, Nishio H, Shirakawa T, Kawamura T, Tanaka R, Nakamura H, Iijima K, Yoshikawa N. Polymorphisms of renin-angiotensin system genes in childhood IgA nephropathy. Pediatric nephrology (Berlin, Germany). 2001; 16:350-5.
74. Hahn H, Ku SE, Kim KS, Park YS, Yoon CH, Cheong HI. Implication of genetic variations in congenital obstructive nephropathy. Pediatric nephrology (Berlin, Germany). 2005; 20:1541-4. doi: 10.1007/s00467-005-1999-1.
75. Frishberg Y, Becker-Cohen R, Halle D, Feigin E, Eisenstein B, Halevy R, Lotan D, Juabeh I, Ish-Shalom N, Magen D, ShvilY, Sinai-Treiman L, Drukker A. Genetic polymorphisms of the renin-angiotensin system and the outcome of focal segmental glomerulosclerosis in children. Kidney Int. 1998; 54:1843-9. doi: 10.1046/j.1523-1755.1998.00218.x.
76. Pardo R, Malaga S, Coto E, Navarro M, Alvarez V, Espinosa L, Alvarez R, Vallo A, Loris C, Braga S. Renin-angiotensin system polymorphisms and renal scarring. Pediatric nephrology (Berlin, Germany). 2003; 18:110-4. doi: 10.1007/s00467-002-1031-y..
77. Liu KP, Lin CY, Chen HJ, Wei CF, Lee-Chen GJ. Renin-angiotensin system polymorphisms in Taiwanese primary vesicoureteral reflux. Pediatric nephrology (Berlin, Germany). 2004; 19:594-601. doi: 10.1007/s00467-003-1379-7.
78. Papp F, Friedman AL, Bereczki C, Haszon I, Kiss E, Endreffy E, Turi S. Renin-angiotensin gene polymorphism in children with uremia and essential hypertension. Pediatric nephrology (Berlin, Germany). 2003; 18:150-4. doi: 10.1007/s00467-002-1032-x.
79. Akman B, Tarhan C, Arat Z, Sezer S, Ozdemir FN. Renin-angiotensin system polymorphisms: a risk factor for progression to end-stage renal disease in vesicoureteral reflux patients. Renal failure. 2009; 31:196-200. doi: 10.1080/08860220802669826.
80. Hunley TE, Julian BA, Phillips JA 3rd, Summar ML, Yoshida H, Horn RG, Brown NJ, Fogo A, Ichikawa I, Kon V. Angiotensin converting enzyme gene polymorphism: potential silencer motif and impact on progression in IgA nephropathy. Kidney Int. 1996; 49:571-7.
81. Rodriguez-Perez JC, Macias-Reyes A, Jimenez-Sosa A, Companioni O, Rodriguez-Esparragon FJ, Cobo MA, Checa-Andres MD, Palop-Cubillo L, Alonso A, Torres A. A synergistic association of ACE I/D and eNOS G894T gene variants with the progression of immunoglobulin A nephropathy - a pilot study. Am J Nephrol. 2009; 30:303-9. doi: 10.1159/000225938. P
82. Rogus JJ, Moczulski D, Freire MBS, Yang YD, Warram JH, Krolewski AS. Diabetic nephropathy is associated with AGT polymorphism T235 - Results of a family-based study. Hypertension. 1998; 31:627-31. doi: 10.1161/01.hyp.31.2.627.
83. Solini A, Giacchetti G, Sfriso A, Fioretto P, Sardu C, Saller A, Tonolo G, Maioli M, Mantero F, Nosadini R. Polymorphisms of angiotensin-converting enzyme and angiotensinogen genes in type 2 diabetic sibships in relation to albumin excretion rate. Am J Kidney Dis. 1999; 34:1002-9. doi: 10.1016/s0272-6386(99)70004-1.
84. Frimat L, Philippe C, Maghakian MN, Jonveaux P, Hurault de Ligny B, Guillemin F, Kessler M. Polymorphism of angiotensin converting enzyme, angiotensinogen, and angiotensin II type 1 receptor genes and end-stage renal failure in IgA nephropathy: IGARAS--a study of 274 Men. J Am Soc Nephrol. 2000; 11:2062-7.
85. Lee KB, Kim UK. Angiotensinogen and angiotensin II type 1 receptor gene polymorphism in patients with autosomal dominant polycystic kidney disease: effect on hypertension and ESRD. Yonsei medical journal. 2003; 44:641-7.
86. Su SL, Yang HY, Wu CC, Lee HS, Lin YF, Hsu CA, Lai CH, Lin C, Kao SY, Lu KC. Gene-gene interactions in renin-angiotensin-aldosterone system contributes to end-stage renal disease susceptibility in a Han Chinese population. ScientificWorldJournal. 2014; 2014:169798. doi: 10.1155/2014/169798..
87. Su SL, Lu KC, Lin YF, Hsu YJ, Lee PY, Yang HY, Kao SY. Gene polymorphisms of angiotensin-converting enzyme and angiotensin II type 1 receptor among chronic kidney disease patients in a Chinese population. J Renin Angiotensin Aldosterone Syst. 2012; 13:148-54. doi: 10.1177/1470320311430989.
88. Luther Y, Bantis C, Ivens K, Fehsel K, Kolb-Bachhofen V, Heering P. Effects of the genetic polymorphisms of the renin-angiotensin system on focal segmental glomerulosclerosis. Kidney & blood pressure research. 2003; 26:333-7. doi: 73939.
89. Bantis C, Ivens K, Kreusser W, Koch M, Klein-Vehne N, Grabensee B, Heering P. Influence of genetic polymorphisms of the renin-angiotensin system on IgA nephropathy. Am J Nephrol. 2004; 24:258-67. doi: 10.1159/000077398.
90. Doria A, Onuma T, Gearin G, Freire MB, Warram JH, Krolewski AS. Angiotensinogen polymorphism M235T, hypertension, and nephropathy in insulin-dependent diabetes. Hypertension. 1996; 27:1134-9.
91. Osawa N, Koya D, Araki S, Uzu T, Tsunoda T, Kashiwagi A, Nakamura Y, Maeda S. Combinational effect of genes for the renin-angiotensin system in conferring susceptibility to diabetic nephropathy. J Hum Genet. 2007; 52:143-51. doi: 10.1007/s10038-006-0090-5.
92. Tomino Y, Makita Y, Shike T, Gohda T, Haneda M, Kikkawa R, Watanabe T, Baba T, Yoshida H. Relationship between polymorphism in the angiotensinogen, angiotensin-converting enzyme or angiotensin II receptor and renal progression in Japanese NIDDM patients. Nephron. 1999; 82:139-44.
93. Anbazhagan K, Sampathkumar K, Ramakrishnan M, Gomathi P, Gomathi S, Selvam GS. Analysis of polymorphism in renin angiotensin system and other related genes in South Indian chronic kidney disease patients. Clinica chimica acta. 2009; 406:108-12. doi: 10.1016/j.cca.2009.06.003.
94. Buraczynska M, Ksiazek P, Drop A, Zaluska W, Spasiewicz D, Ksiazek A. Genetic polymorphisms of the renin-angiotensin system in end-stage renal disease. Nephrol Dial Transplant. 2006; 21:979-83. doi: 10.1093/ndt/gfk012.
95. Chang HR, Cheng CH, Shu KH, Chen CH, Lian JD, Wu MY. Study of the polymorphism of angiotensinogen, anigiotensin-converting enzyme and angiotensin receptor in type II diabetes with end-stage renal disease in Taiwan. Journal of the Chinese Medical Association. 2003; 66:51-6.
96. Chen WJ, Huang YL, Shiue HS, Chen TW, Lin YF, Huang CY, LinYC, Han BC, Hsueh YM. Renin-angiotensin-aldosterone system related gene polymorphisms and urinary total arsenic is related to chronic kidney disease. Toxicology and applied pharmacology. 2014; 279:95-102. doi: 10.1016/j.taap.2014.05.011.
97. Eroglu Z, Cetinkalp S, Erdogan M, Kosova B, Karadeniz M, Kutukculer A, Gunduz C, Tetik A, Topcuoglu N, Ozgen AG, Tuzen M. Association of the angiotensinogen M235T and angiotensin-converting enzyme insertion/deletion gene polymorphisms in Turkish type 2 diabetic patients with and without nephropathy. J Diabetes Complications. 2008; 22:186-90. doi: 10.1016/j.jdiacomp.2006.12.004.
98. Fabris B, Bortoletto M, Candido R, Barbone F, Cattin MR, Calci M, Scanferla F, Tizzoni L, Giacca M, Carretta R. Genetic polymorphisms of the renin-angiotensin-aldosterone system and renal insufficiency in essential hypertension. J Hypertens. 2005; 23:309-16.
99. Fogarty DG, Harron JC, Hughes AE, Nevin NC, Doherty CC, Maxwell AP. A Molecular Variant of Angiotensinogen Is Associated With Diabetic Nephropathy in IDDM. Diabetes. 1996; 45:1204-8. doi: 10.2337/diab.45.9.1204.
100. Fradin S, Goulet-Salmon B, Chantepie M, Grandhomme F, Morello R, Jauzac P, Reznik Y. Relationship between polymorphisms in the renin-angiotensin system and nephropathy in type 2 diabetic patients. Diabetes & metabolism. 2002; 28:27-32.
101. Freire MB, Ji L, Onuma T, Orban T, Warram JH, Krolewski AS. Gender-specific association of M235T polymorphism in angiotensinogen gene and diabetic nephropathy in NIDDM. Hypertension. 1998; 31:896-9.
102. Gao J, Yu QL, Fu RG, Wei LT, Wang M, Dong FM, Wang Z, Yang PT, Liu XH, Dai ZJ. Lack of Association Between Polymorphisms in AGT and ATR1 and IgA Nephropathy in a Chinese Population. Genetic testing and molecular biomarkers. 2015; 19:710-3. doi: 10.1089/gtmb.2015.0167.
103. Huang HD, Lin FJ, Li XJ, Wang LR, Jiang GR. Genetic polymorphisms of the renin-angiotensin-aldosterone system in Chinese patients with end-stage renal disease secondary to IgA nephropathy. Chinese medical journal. 2010; 123:3238-42.
104. Kim SM, Chin HJ, Oh YK, Kim YS, Kim S, Lim CS. Blood pressure-related genes and the progression of IgA nephropathy. Nephron Clinical practice. 2009; 113:c301-8. doi: 10.1159/000235948.
105. Losito A, Kalidas K, Santoni S, Ceccarelli L, Jeffery S. Polymorphism of renin-angiotensin system genes in dialysis patients--association with cerebrovascular disease. Nephrol Dial Transplant. 2002; 17:2184-8.
106. Marre M, Jeunemaitre X, Gallois Y, Rodier M, Chatellier G, Sert C, Dusselier L, Kahal Z, Chaillous L, Halimi S, Muller A, Sackmann H, Bauduceau B, Bled F, Passa P, Alhenc-Gelas F. Contribution of genetic polymorphism in the renin-angiotensin system to the development of renal complications in insulin-dependent diabetes: Genetique de la Nephropathie Diabetique (GENEDIAB) study group. The Journal of clinical investigation. 1997; 99:1585-95. doi: 10.1172/jci119321.
107. Pawlik M, Mostowska A, Lianeri M, Oko A, Jagodzinski PP. Association of aldosterone synthase (CYP11B2) gene -344T/C polymorphism with the risk of primary chronic glomerulonephritis in the Polish population. J Renin Angiotensin Aldosterone Syst. 2014; 15:553-8. doi: 10.1177/1470320313489588.
108. Pei Y, Scholey J, Thai K, Suzuki M, Cattran D. Association of angiotensinogen gene T235 variant with progression of immunoglobin A nephropathy in Caucasian patients. The Journal of clinical investigation. 1997; 100:814-20. doi: 10.1172/jci119596.
109. Ringel J, Beige J, Kunz R, Distler A, Sharma AM. Genetic variants of the renin-angiotensin system, diabetic nephropathy and hypertension. Diabetologia. 1997; 40:193-9. doi: 10.1007/s001250050662.
110. Schmidt S, Giessel R, Bergis KH, Strojek K, Grzeszczak W, Ganten D, Rits E. Angiotensinogen gene M235T polymorphism is not associated with diabetic nephropathy. The Diabetic Nephropathy Study Group. Nephrol Dial Transplant. 1996; 11:1755-61.
111. Shaikh R, Shahid SM, Mansoor Q, Ismail M, Azhar A. Genetic variants of ACE (Insertion/Deletion) and AGT (M268T) genes in patients with diabetes and nephropathy. J Renin Angiotensin Aldosterone Syst. 2014; 15:124-30. doi: 10.1177/1470320313512390.
112. Stratta P, Bermond F, Guarrera S, Canavese C, Carturan S, Dall'Omo A, Ciccone G, Bertola L, Mazzola G, Fasano E, Matullo G. Interaction between gene polymorphisms of nitric oxide synthase and renin-angiotensin system in the progression of membranous glomerulonephritis. Nephrol Dial Transplant. 2004; 19:587-95.
113. Tarnow L, Cambien F, Rossing P, Nielsen FS, Hansen BV, Ricard S, Poirier O, Paraving HH. Angiotensinogen gene polymorphisms in IDDM patients with diabetic nephropathy. Diabetes. 1996; 45:367-9.
114. Frans J. van Ittersum AMEdM, Sandra Thijssen, Peter de Knijff, Eline Slagboom, Yvo Smulders, Lise Tarno3, Ab J. M. Donke1, Henk J. G. Bil4 and Coen D. A. Stehouwer. Genetic polymorphisms of the renin–angiotensin system and complications of insulin-dependent diabetes mellitus. Nephrol Dial Transplant. 2000.
115. Wang J, Zhu X, Yang L, Liu Y, Zhou W, Li H. Relationship between angiotensinogen gene M235T variant with diabetic nephropathy in Chinese NIDDM. Chinese medical journal. 1999; 112:797-800.
116. Woo KT, Lau YK, Choong LH, Zhao Y, Tan HB, Fook-Chong S, Tan EK, Yap HK, Wong KS. Polymorphism of renin-angiotensin system genes in IgA nephropathy. Nephrology (Carlton). 2004; 9:304-9. doi: 10.1111/j.1440-1797.2004.00291.x.
117. Wu S, Xiang K, Zheng T, Sun D, Weng Q, Zhao H, Li J. Relationship between the renin-angiotensin system genes and diabetic nephropathy in the Chinese. Chinese medical journal. 2000; 113:437-41.
118. Zsom M, Fulop T, Zsom L, Barath A, Maroti Z, Endreffy E. Genetic polymorphisms and the risk of progressive renal failure in elderly Hungarian patients. Hemodialysis international International Symposium on Home Hemodialysis. 2011; 15:501-8. doi: 10.1111/j.1542-4758.2011.00593.x.
119. Zychma MJ, Zukowska-Szczechowska E, Lacka BI, Grzeszczak W. Angiotensinogen M235T and chymase gene CMA/B polymorphisms are not associated with nephropathy in type II diabetes. Nephrology Dialysis Transplantation. 2000; 15:1965-70. doi: 10.1093/ndt/15.12.1965.
120. Ahluwalia TS, Ahuja M, Rai TS, Kohli HS, Bhansali A, Sud K,Khullar M. ACE variants interact with the RAS pathway to confer risk and protection against type 2 diabetic nephropathy. DNA and cell biology. 2009; 28:141-50. doi: 10.1089/dna.2008.0810.
121. McLaughlin KJ, Jagger C, Small M, Jardine AG. Effect of angiotensinogen gene T235 variant on the development of diabetic complications in type II diabetes mellitus. Lancet. 1995; 346:1160. doi: 10.1016/s0140-6736(95)91831-0.
122. Filler G, Yang F, Martin A, Stolpe J, Neumayer H-H, Hocher B. Renin angiotensin system gene polymorphisms in pediatric renal transplant recipients. Pediatric Transplantation. 2001; 5:166-73. doi: 10.1034/j.1399-3046.2001.00053.x.
123. Ayed K, Ayed-Jendoubi S, Ben Abdallah T, Bardi R, Gorgi Y, Sfar I, Dhrif B, Abderrahim E, Kheder A. Polymorphism of the renin-angiotensin-aldosterone system in patients with chronic allograft dysfunction. Transpl Immunol. 2006; 15:303-9. doi: 10.1016/j.trim.2005.09.011.
124. Gumprecht J, Zychma MJ, Grzeszczak W, Zukowska-Szczechowska E. Angiotensin I-converting enzyme gene insertion/deletion and angiotensinogen M235T polymorphisms: risk of chronic renal failure. End-Stage Renal Disease Study Group. Kidney Int. 2000; 58:513-9. doi: 10.1046/j.1523-1755.2000.00197.x.
125. Tien KJ, Hsiao JY, Hsu SC, Liang HT, Lin SR, Chen HC, Hsieh MC. Gender-dependent effect of ACE I/D and AGT M235T polymorphisms on the progression of urinary albumin excretion in Taiwanese with type 2 diabetes. Am J Nephrol. 2009; 29:299-308. doi: 10.1159/000163592.
126. Lau YK, Woo KT, Choong HL, Zhao Y, Tan HB, Chong SM, Tan EK, Yap HK, Wong KS. Renin-angiotensin system gene polymorphisms: its impact on IgAN and its progression to end-stage renal failure among Chinese in Singapore. Nephron Physiol. 2004; 97:p1-8. doi: 10.1159/000077596.
127. Chowdhury TA, Dronsfield MJ, Kumar S, Gough SLC, Gibson SP, Khatoon A, MacDonald F, Rowe BR, Dunger DB, Dean JD, Davies SJ, Webber J, Smith PR, et al . Examination of two genetic polymorphisms within the renin-angiotensin system: no evidence for an association with nephropathy in IDDM. Diabetologia. 1996; 39:1108-14. doi: 10.1007/bf00400661.
128. Mollsten A, Kockum I, Svensson M, Rudberg S, Ugarph-Morawski A, Brismar K, Eriksson JW, Dahlquist G. The effect of polymorphisms in the renin-angiotensin-aldosterone system on diabetic nephropathy risk. J Diabetes Complications. 2008; 22:377-83. doi: 10.1016/j.jdiacomp.2007.06.005.
129. Dudley CRK, Keavney B, Stratton IM, Turner RC, Ratcliffe PJ. U.K. Prospective Diabetes Study XV: Relationship of renin-angiotensin system gene polymorphisms with microalbuminuria in NIDDM. Kidney International. 1995; 48:1907-11. doi: 10.1038/ki.1995.490.
130. Lovati E, Richard A, Frey BM, Frey FJ, Ferrari P. Genetic polymorphisms of the renin-angiotensin-aldosterone system in end-stage renal disease. Kidney Int. 2001; 60:46-54. doi: 10.1046/j.1523-1755.2001.00769.x.
131. Miura J, Uchigata Y, Yokoyama H, Omori Y, Iwamoto Y. Genetic polymorphism of renin-angiotensin system is not associated with diabetic vascular complications in Japanese subjects with long-term insulin dependent diabetes mellitus. Diabetes Research and Clinical Practice. 1999; 45:41-9. doi: 10.1016/s0168-8227(99)00059-5.
132. Prasad P, Tiwari AK, Kumar KM, Ammini AC, Gupta A, Gupta R, Sharma AK, Rao AR, Nagendra R, Chandra TS, Tiwari SC, Rastogi, P, Gupta BL, Thelma BK. Chronic renal insufficiency among Asian Indians with type 2 diabetes: I. Role of RAAS gene polymorphisms. BMC Med Genet. 2006; 7:42. doi: 10.1186/1471-2350-7-42.
133. El-Essawy AB, Berthoux P, Cécillon S, C.Deprèle, Thibaudin D, Filippis JPD, E Alamartine, Berthoux F. Hypertension after renal transplantation and polymorphism of genes involved in essential hypertension: ACE, AGT, AT1R and ecNOS. Clinical Nephrology. 2002; 57:192-200. doi: 10.5414/cnp57192.
