# Supplementary material for: Angiotensin II receptor type 1 A1166C modifies the association between angiotensinogen M235T and chronic kidney disease
Source: Oncotarget. 2017 Oct 26;8(64):107833–43. doi: 10.18632/oncotarget.22121 (PMC5746107; doi:10.18632/oncotarget.22121)
Supplement: Supplementary file 4 [file oncotarget-08-107833-s004.docx]

**Supplementary Table 3: Summary of studies included in the meta-analysis**

| **Author** | **Year** | **Country** | **Ethnicity** | **Study design^a^** | **CKD type^b^** | **Kidney function of case** ^c^ | **Definition of case group** ^d^ |
| --- | --- | --- | --- | --- | --- | --- | --- |
| This Study |  | Taiwan | Asian | CC | Mixed | ESRD | Dialysis patient |
| Gao [1] | 2015 | China | Asian | CC | IgAN | non-ESRD | histologically biopsy |
| Sarkar [2] | 2015 | India | Asian | CC | Mixed | ESRD | Dialysis patient |
| Chen [3] | 2014 | Taiwan | Asian | CC | Mixed | non-ESRD | eGFR < 60 ml/min/1.73 m2 |
| Pawlik [4]  Shaikh [5] | 2014  2014 | Poland  Pakistan | Caucasian  Caucasian | CC  CC | Mixed  DN | non-ESRD  non-ESRD | histologically biopsy  diabetic nephropathy |
| Zsom [6] | 2011 | Hungary | Caucasian | CC | Mixed | non-ESRD | histologically biopsy & eGFR < 60 ml/min/1.73 m^2^ |
| Huang [7] | 2010 | China | Asian | CC | IgAN | non-ESRD | histologically biopsy |
| Ahluwalia [8] | 2009 | India | Asian | CC | DN | non-ESRD | AER>200ug/min or ACR>300mg/g |
| Anbazhagan [9] | 2009 | India | Asian | CC | Mixed | ESRD | Dialysis patient |
| Kim [10] | 2009 | Korea | Asian | CC | IgAN | non-ESRD | histologically biopsy |
| Eroglu [11] | 2008 | Turkey | Caucasian | CS | DN | non-ESRD | UAE >30 mg/day |
| Buraczynska [12] | 2006 | Poland | Caucasian | CC | Mixed | ESRD | HD |
| Prasad [13] | 2006 | India | Asian | CS | DN | non-ESRD | SCr > 1.5 mg/dL or AER>200mg/L or RTN |
| Fabris [14] | 2005 | Italy | Caucasian | CC | HN | non-ESRD | SCr > 1.5 mg/dL or CT |
| Stratta [15] | 2004 | Italy | Caucasian | CC | GN | non-ESRD | CT &s-CR<1.5mg/dL |
| Woo [16] | 2004 | Singapore | Asian | CC | IgAN | non-ESRD | CT & UAE >1g/day |
| Chang [17] | 2003 | Taiwan | Asian | CC | CC | ESRD | Dialysis patient |
| El-Essawy [18] | 2002 | France | Caucasian | CS | Mixed | ESRD | RRT |
| Fradin [19] | 2002 | France | Caucasian | CS | DN | non-ESRD | UAE >30 mg/day or > 20 μg/min |
| Losito [20] | 2002 | United Kingdom | Caucasian | CC | Mixed | ESRD | HD |
| Lovati [21] | 2001 | Switzerland | Caucasian | CC | Mixed | ESRD | Dialysis patient |
| Thomas [22] | 2001 | Hong Kong | Asian | CS | DN | non-ESRD | UAE > 20 μg/min |
| van Ittersum [23] | 2000 | Netherlands | Caucasian | CS | DN | non-ESRD | UAE >30 mg/day |
| Wu [24] | 2000 | China | Asian | CS | DN | non-ESRD | no description |
| Zychma [25] | 2000 | Poland | Caucasian | CC | DN | non-ESRD | no description |
| Miura [26] | 1999 | Japan | Asian | CS | DN | non-ESRD | UAE > 10 μg/min |
| Wang [27] | 1999 | China | Asian | CC | DN | non-ESRD | UAE > 20 μg/min |
| Freire [28] | 1999 | United States | Caucasian | CC | DN | non-ESRD | ACR> 250 mg/g (men) or > 355 mg/g (women) |
| Marre [29] | 1997 | France | Caucasian | CS | DN | non-ESRD | UAE > 30 mg/day |
| Pei [30] | 1997 | Canada | Caucasian | CC | IgAN | non-ESRD | histologically biopsy |
| Ringel [31] | 1997 | Germany | Caucasian | CS | Mixed | non-ESRD | UAE >30 mg/day |
| Fogarty [32] | 1996 | United Kingdom | Caucasian | CC | DN | non-ESRD | UAE >300 mg/day |
| Schmidt [33] | 1996 | Germany | Caucasian | CC | DN | non-ESRD | UAE >30 mg/day |
| Tarnow [34] | 1996 | Denmark | Caucasian | CC | DN | non-ESRD | no description |

^a^: CC – case control study; CS – cross sectional study.

^b^: IgAN – IgA nephropathy; DN – diabetic nephropathy; GN –glomerulonephritis; Mixed – combine.

^c^: ESRD – only ESRD patients; non-ESRD – not only ESRD patients.

^d^: ACR – Albumin creatinine ratio; AER – Albumin excretion rate; UAE – urinary albumin excretion rate; eGFR – estimated glomerular filtration rate; HD – hemodialysis; CCr – creatinine clearance; RRT – renal replacement therapy; CT – computed tomography; SCr – serum creatinine.

**Supplementary Table 3: Summary of studies included in the meta-analysis (continues)**

| **Author** | **year** | **quality** | **age** | **sex** | **BMI** | **DM** | **HT** | **MM_case** | **MT_case** | **TT_case** | **MM_control** | **MT_control** | **TT_control** |
| --- | --- | --- | --- | --- | --- | --- | --- | --- | --- | --- | --- | --- | --- |
| This Study |  | 5 | 64.5 | 46.7 | 22.4 |  | 57.8 | 560 | 67 | 7 | 656 | 72 | 10 |
| Gao | 2015 | 2 | 32 | 65.2 |  | 100 | 49.3 | 225 | 110 | 16 | 192 | 108 | 10 |
| Sarkar | 2015 | 5 | 51.3 | 70.5 | 24.2 | 60.1 | 39.9 | 42 | 97 | 34 | 79 | 149 | 101 |
| Chen | 2014 | 6 | 65.1 | 61.4 |  | 39.1 | 55.8 | 4 | 65 | 164 | 13 | 124 | 312 |
| Pawlik | 2014 | 5 | 39.2 | 57.1 |  |  |  | 30 | 81 | 29 | 41 | 97 | 49 |
| Shaikh | 2014 | 2 | 55.9 |  |  | 100 | 88.9 | 1 | 47 | 62 | 3 | 77 | 35 |
| Zsom | 2011 | 5 | 66 | 57.6 |  |  |  | 85 | 158 | 66 | 50 | 100 | 50 |
| Huang | 2010 | 4 | 40 | 0.5 |  |  | 53.8 | 2 | 31 | 97 | 1 | 38 | 81 |
| Ahluwalia | 2009 | 6 | 58 | 66.2 | 23.6 | 100 | 58.7 | 54 | 104 | 82 | 108 | 120 | 27 |
| Anbazhagan | 2009 | 4 | 49 | 72.9 |  | 27.5 | 74.9 | 11 | 51 | 39 | 29 | 54 | 14 |
| Kim(1) | 2009 | 5 | 32.4 | 100 |  |  | 55.6 | 3 | 37 | 77 | 7 | 35 | 100 |
| Kim(2) | 2009 | 5 | 35 | 0 |  |  | 62.5 | 4 | 28 | 64 | 6 | 54 | 98 |
| Eroglu | 2008 | 5 | 58 | 41.3 | 28.9 | 100 | 37.6 | 12 | 24 | 10 | 15 | 32 | 9 |
| Buraczynska | 2006 | 5 | 51 | 55.7 |  | 19 | 78.2 | 190 | 354 | 201 | 163 | 238 | 119 |
| Prasad | 2006 | 4 | 57 | 33.2 |  | 100 |  | 31 | 82 | 82 | 95 | 86 | 45 |
| Fabris | 2005 | 5 | 60 | 77.9 | 25.9 | 0 | 100 | 22 | 34 | 30 | 61 | 74 | 37 |
| Stratta | 2004 | 6 | 50 | 66.7 |  |  | 39.3 | 39 | 58 | 20 | 46 | 90 | 35 |
| Woo | 2004 | 4 | 43 | 47.5 |  |  | 36.4 | 2 | 23 | 93 | 1 | 29 | 64 |
| Chang | 2003 | 5 | 62 | 48 |  | 100 |  | 1 | 39 | 89 | 0 | 22 | 94 |
| El-Essawy | 2002 | 5 | 42.2 | 33.3 |  |  |  | 93 | 144 | 56 | 62 | 84 | 35 |
| Fradin | 2002 | 5 | 57 | 53 | 31.5 | 100 |  | 48 | 44 | 25 | 40 | 59 | 19 |
| Losito | 2002 | 5 | 67 | 60.6 | 24.9 | 13.7 | 63.1 | 46 | 73 | 42 | 51 | 88 | 30 |
| Lovati | 2001 | 3 | 54.3 | 56.9 | 24.9 | 12.3 | 50.7 | 80 | 139 | 41 | 120 | 153 | 54 |
| Thomas(1) | 2001 | 5 | 43.6 | 30.8 | 27.5 | 0 | 100 | 2 | 4 | 20 | 2 | 18 | 52 |
| Thomas(2) | 2001 | 5 | 50.5 | 43 | 26.1 | 100 | 62.1 | 4 | 36 | 110 | 5 | 69 | 181 |
| van Ittersum | 2000 | 5 | 55 | 59.4 |  | 100 | 79.7 | 19 | 39 | 11 | 71 | 96 | 21 |
| Wu | 2000 | 5 | 60 | 54.9 |  | 100 |  | 2 | 17 | 52 | 7 | 12 | 22 |
| Zychma | 2000 | 4 | 62 |  | 29.2 | 100 | 68.7 | 116 | 228 | 106 | 63 | 116 | 64 |
| Miura | 1999 | 5 | 35.7 | 33.7 |  | 100 | 20.9 | 3 | 34 | 61 | 2 | 32 | 69 |
| Wang | 1999 | 6 | 58 | 57.3 | 24.1 | 100 | 73.8 | 5 | 24 | 67 | 12 | 34 | 38 |
| Freire | 1998 | 5 | 62 | 62 |  | 100 | 66 | 45 | 44 | 26 | 44 | 60 | 14 |
| Marre | 1997 | 7 | 43 | 57.3 | 23.5 | 100 | 57.1 | 109 | 154 | 74 | 49 | 78 | 29 |
| Pei | 1997 | 5 |  |  |  |  |  | 60 | 79 | 29 | 34 | 50 | 16 |
| Ringel(1) | 1997 | 6 | 38.9 | 56.7 | 24.3 | 100 | 60 | 42 | 61 | 31 | 71 | 115 | 40 |
| Ringel(2) | 1997 | 6 | 61.4 | 52.2 | 27.8 | 100 | 23 | 46 | 88 | 67 | 45 | 62 | 33 |
| Fogarty | 1996 | 5 | 44.6 | 70.5 |  | 100 |  | 37 | 40 | 18 | 37 | 55 | 8 |
| Schmidt(1) | 1996 | 6 | 44 | 59.4 | 25.1 | 100 | 57.5 | 49 | 101 | 30 | 67 | 139 | 37 |
| Schmidt(2) | 1996 | 6 | 65 | 50 | 28.6 | 100 | 76 | 102 | 145 | 63 | 112 | 170 | 71 |
| Tarnow | 1996 | 5 | 41 | 62.1 | 24.1 | 100 | 68.4 | 73 | 97 | 25 | 67 | 95 | 23 |

**Supplementary Table 3: Summary of studies included in the meta-analysis (continues)**

| **Author** | **year** | **MM_case** | **MT_case** | **TT_case** | **MM_control** | **MT_control** | **TT_control** | **AA_case** | **AC_case** | **CC_case** | **AA_control** | **AC_control** | **CC_control** |
| --- | --- | --- | --- | --- | --- | --- | --- | --- | --- | --- | --- | --- | --- |
| This Study |  | 13 | 168 | 453 | 37 | 205 | 497 | 560 | 67 | 7 | 656 | 72 | 10 |
| Gao | 2015 | 225 | 110 | 16 | 192 | 108 | 10 | 3 | 42 | 306 | 0 | 38 | 272 |
| Sarkar | 2015 | 42 | 97 | 34 | 79 | 149 | 101 |  |  |  |  |  |  |
| Chen | 2014 | 4 | 65 | 164 | 13 | 124 | 312 | 208 | 25 | 0 | 407 | 42 | 0 |
| Pawlik | 2014 | 30 | 81 | 29 | 41 | 97 | 49 |  |  |  |  |  |  |
| Shaikh | 2014 | 1 | 47 | 62 | 3 | 77 | 35 |  |  |  |  |  |  |
| Zsom | 2011 | 85 | 158 | 66 | 50 | 100 | 50 | 167 | 125 | 16 | 100 | 88 | 12 |
| Huang | 2010 | 2 | 31 | 97 | 1 | 38 | 81 | 113 | 17 | 0 | 100 | 20 | 0 |
| Ahluwalia | 2009 | 54 | 104 | 82 | 108 | 120 | 27 | 104 | 112 | 24 | 131 | 119 | 5 |
| Anbazhagan | 2009 | 11 | 51 | 39 | 29 | 54 | 14 |  |  |  |  |  |  |
| Kim(1) | 2009 | 3 | 37 | 77 | 7 | 35 | 100 | 114 | 16 | 0 | 132 | 10 | 0 |
| Kim(2) | 2009 | 4 | 28 | 64 | 6 | 54 | 98 | 100 | 8 | 0 | 144 | 14 | 0 |
| Eroglu | 2008 | 12 | 24 | 10 | 15 | 32 | 9 |  |  |  |  |  |  |
| Buraczynska | 2006 | 190 | 354 | 201 | 163 | 238 | 119 | 346 | 322 | 77 | 322 | 182 | 16 |
| Prasad | 2006 | 31 | 82 | 82 | 95 | 86 | 45 | 169 | 25 | 2 | 194 | 29 | 2 |
| Fabris | 2005 | 22 | 34 | 30 | 61 | 74 | 37 | 40 | 42 | 4 | 106 | 59 | 7 |
| Stratta | 2004 | 39 | 58 | 20 | 46 | 90 | 35 | 62 | 43 | 12 | 90 | 67 | 14 |
| Woo | 2004 | 2 | 23 | 93 | 1 | 29 | 64 | 110 | 7 | 1 | 84 | 10 | 0 |
| Chang | 2003 | 1 | 39 | 89 | 0 | 22 | 94 |  |  |  |  |  |  |
| El-Essawy | 2002 | 93 | 144 | 56 | 62 | 84 | 35 | 150 | 125 | 19 | 101 | 63 | 17 |
| Fradin | 2002 | 48 | 44 | 25 | 40 | 59 | 19 | 74 | 31 | 12 | 61 | 52 | 5 |
| Losito | 2002 | 46 | 73 | 42 | 51 | 88 | 30 | 72 | 72 | 16 | 91 | 64 | 14 |
| Lovati | 2001 | 80 | 139 | 41 | 120 | 153 | 54 |  |  |  |  |  |  |
| Thomas(1) | 2001 | 2 | 4 | 20 | 2 | 18 | 52 | 24 | 2 | 0 | 64 | 8 | 0 |
| Thomas(2) | 2001 | 4 | 36 | 110 | 5 | 69 | 181 | 139 | 11 | 0 | 232 | 23 | 0 |
| van Ittersum | 2000 | 19 | 39 | 11 | 71 | 96 | 21 | 30 | 31 | 8 | 70 | 100 | 19 |
| Wu | 2000 | 2 | 17 | 52 | 7 | 12 | 22 | 56 | 15 | 0 | 33 | 8 | 0 |
| Zychma | 2000 | 116 | 228 | 106 | 63 | 116 | 64 |  |  |  |  |  |  |
| Miura | 1999 | 3 | 34 | 61 | 2 | 32 | 69 |  |  |  |  |  |  |
| Wang | 1999 | 5 | 24 | 67 | 12 | 34 | 38 |  |  |  |  |  |  |
| Freire | 1998 | 45 | 44 | 26 | 44 | 60 | 14 |  |  |  |  |  |  |
| Marre | 1997 | 109 | 154 | 74 | 49 | 78 | 29 | 177 | 141 | 19 | 75 | 64 | 14 |
| Pei | 1997 | 60 | 79 | 29 | 34 | 50 | 16 | 82 | 73 | 13 | 56 | 37 | 7 |
| Ringel(1) | 1997 | 42 | 61 | 31 | 71 | 115 | 40 |  |  |  |  |  |  |
| Ringel(2) | 1997 | 46 | 88 | 67 | 45 | 62 | 33 |  |  |  |  |  |  |
| Fogarty | 1996 | 37 | 40 | 18 | 37 | 55 | 8 |  |  |  |  |  |  |
| Schmidt(1) | 1996 | 49 | 101 | 30 | 67 | 139 | 37 |  |  |  |  |  |  |
| Schmidt(2) | 1996 | 102 | 145 | 63 | 112 | 170 | 71 |  |  |  |  |  |  |
| Tarnow | 1996 | 73 | 97 | 25 | 67 | 95 | 23 |  |  |  |  |  |  |

**REFERENCES**

1. Gao J, Yu QL, Fu RG, Wei LT, Wang M, Dong FM, Wang, Z., Yang PT, Liu XH, Dai ZJ. Lack of Association Between Polymorphisms in AGT and ATR1 and IgA Nephropathy in a Chinese Population. Genetic testing and molecular biomarkers. 2015; 19:710-3. doi: 10.1089/gtmb.2015.0167.

2. Sarkar S, Gupta V, Kumar A, Chaudhary M, Diyundi S, Sehajpal PK, Thangaraj K, Rajender S. M235T polymorphism in the AGT gene and A/G(I8-83) substitution in the REN gene correlate with end-stage renal disease. Nephron. 2015; 129:104-8. doi: 10.1159/000370074.

3. Chen WJ, Huang YL, Shiue HS, Chen TW, Lin YF, Huang CY, Lin YC, Han BC, Hsueh YM. Renin-angiotensin-aldosterone system related gene polymorphisms and urinary total arsenic is related to chronic kidney disease. Toxicology and applied pharmacology. 2014; 279:95-102. doi: 10.1016/j.taap.2014.05.011.

4. Pawlik M, Mostowska A, Lianeri M, Oko A, Jagodzinski PP. Association of aldosterone synthase (CYP11B2) gene -344T/C polymorphism with the risk of primary chronic glomerulonephritis in the Polish population. Journal of the renin-angiotensin-aldosterone system. 2014; 15:553-8. doi: 10.1177/1470320313489588.

5. Shah VN, Cheema BS, Sharma R, Khullar M, Kohli HS, Ahluwalia TS, Mohan V, Bhansali A. ACACbeta gene (rs2268388) and AGTR1 gene (rs5186) polymorphism and the risk of nephropathy in Asian Indian patients with type 2 diabetes. Molecular and cellular biochemistry. 2013; 372:191-8. doi: 10.1007/s11010-012-1460-2.

6. Zsom M, Fulop T, Zsom L, Barath A, Maroti Z, Endreffy E. Genetic polymorphisms and the risk of progressive renal failure in elderly Hungarian patients. Hemodialysis international International Symposium on Home Hemodialysis. 2011; 15:501-8. doi: 10.1111/j.1542-4758.2011.00593.x.

7. Huang HD, Lin FJ, Li XJ, Wang LR, Jiang GR. Genetic polymorphisms of the renin-angiotensin-aldosterone system in Chinese patients with end-stage renal disease secondary to IgA nephropathy. Chinese medical journal. 2010; 123:3238-42.

8. Ahluwalia TS, Ahuja M, Rai TS, Kohli HS, Bhansali A, Sud K, Khullar M. ACE variants interact with the RAS pathway to confer risk and protection against type 2 diabetic nephropathy. DNA and cell biology. 2009; 28:141-50. doi: 10.1089/dna.2008.0810.

9. Anbazhagan K, Sampathkumar K, Ramakrishnan M, Gomathi P, Gomathi S, Selvam GS. Analysis of polymorphism in renin angiotensin system and other related genes in South Indian chronic kidney disease patients. Clinica chimica acta. 2009; 406:108-12. doi: 10.1016/j.cca.2009.06.003.

10. Kim SM, Chin HJ, Oh YK, Kim YS, Kim S, Lim CS. Blood pressure-related genes and the progression of IgA nephropathy. Nephron Clinical practice. 2009; 113:c301-8. doi: 10.1159/000235948.

11. Eroglu Z, Cetinkalp S, Erdogan M, Kosova B, Karadeniz M, Kutukculer A, Gunduz C, Tetik A, Topcuoglu N, Ozgen AG, Tuzun M. Association of the angiotensinogen M235T and angiotensin-converting enzyme insertion/deletion gene polymorphisms in Turkish type 2 diabetic patients with and without nephropathy. Journal of diabetes and its complications. 2008; 22:186-90. doi: 10.1016/j.jdiacomp.2006.12.004.

12. Buraczynska M, Ksiazek P, Drop A, Zaluska W, Spasiewicz D, Ksiazek A. Genetic polymorphisms of the renin-angiotensin system in end-stage renal disease. Nephrology, dialysis, transplantation. 2006; 21:979-83. doi: 10.1093/ndt/gfk012.

13. Prasad P, Tiwari AK, Kumar KM, Ammini AC, Gupta A, Gupta R, Sharma AK, Rao AR, Nagendra R, Chandra TS, Tiwari SC, Rastog P, Gupta BL, Thelma BK. Chronic renal insufficiency among Asian Indians with type 2 diabetes: I. Role of RAAS gene polymorphisms. BMC medical genetics. 2006; 7:42. doi: 10.1186/1471-2350-7-42.

14. Fabris B, Bortoletto M, Candido R, Barbone F, Cattin MR, Calci M, Scanferla F, Tizzoni L, Giacca M, Carretta R. Genetic polymorphisms of the renin-angiotensin-aldosterone system and renal insufficiency in essential hypertension. Journal of hypertension. 2005; 23:309-16.

15. Stratta P, Bermond F, Guarrera S, Canavese C, Carturan S, Dall'Omo A, Ciccone G, Bertola L, Mazzola G, Fasano E, Matullo G. Interaction between gene polymorphisms of nitric oxide synthase and renin-angiotensin system in the progression of membranous glomerulonephritis. Nephrology, dialysis, transplantation. 2004; 19:587-95.

16. Woo KT, Lau YK, Choong LH, Zhao Y, Tan HB, Fook-Chong S, Tan EK, Yap HK, Wong KS. Polymorphism of renin-angiotensin system genes in IgA nephropathy. Nephrology (Carlton, Vic). 2004; 9:304-9. doi: 10.1111/j.1440-1797.2004.00291.x.

17. Chang HR, Cheng CH, Shu KH, Chen CH, Lian JD, Wu MY. Study of the polymorphism of angiotensinogen, anigiotensin-converting enzyme and angiotensin receptor in type II diabetes with end-stage renal disease in Taiwan. Journal of the Chinese Medical Association. 2003; 66:51-6.

18. Basset el EA, Berthoux P, Cecillon S, Deprle C, Thibaudin D, De Filippis JP, Alamartin E, Berthou F. Hypertension after renal transplantation and polymorphism of genes involved in essential hypertension: ACE, AGT, AT1 R and ecNOS. Clinical nephrology. 2002; 57:192-200.

19. Fradin S, Goulet-Salmon B, Chantepie M, Grandhomme F, Morello R, Jauzac P, Reznik Y. Relationship between polymorphisms in the renin-angiotensin system and nephropathy in type 2 diabetic patients. Diabetes & metabolism. 2002; 28:27-32.

20. Losito A, Kalidas K, Santoni S, Ceccarelli L, Jeffery S. Polymorphism of renin-angiotensin system genes in dialysis patients--association with cerebrovascular disease. Nephrology, dialysis, transplantation. 2002; 17:2184-8.

21. Lovati E, Richard A, Frey BM, Frey FJ, Ferrari P. Genetic polymorphisms of the renin-angiotensin-aldosterone system in end-stage renal disease. Kidney international. 2001; 60:46-54. doi: 10.1046/j.1523-1755.2001.00769.x.

22. Thomas GN, Critchley JA, Tomlinson B, Lee ZS, Young RP, Cockran CS, Chan JC. Albuminuria and the renin-angiotensin system gene polymorphisms in type-2-diabetic and in normoglycemic hypertensive Chinese. Clinical nephrology. 2001; 55:7-15.

23. van Ittersum FJ, de Man AM, Thijssen S, de Knijff P, Slagboom E, Smulders Y, Tarnow L, Donker AJ, Bilo HJ, Stehouwer CD. Genetic polymorphisms of the renin-angiotensin system and complications of insulin-dependent diabetes mellitus. Nephrology, dialysis, transplantation. 2000; 15:1000-7.

24. Wu S, Xiang K, Zheng T, Sun D, Weng Q, Zhao H, Li J. Relationship between the renin-angiotensin system genes and diabetic nephropathy in the Chinese. Chinese medical journal. 2000; 113:437-41.

25. Zychma MJ, Zukowska-Szczechowska E, Lacka BI, Grzeszczak W. Angiotensinogen M235T and chymase gene CMA/B polymorphisms are not associated with nephropathy in type II diabetes. Nephrology, dialysis, transplantation. 2000; 15:1965-70.

26. Miura J, Uchigata Y, Yokoyama H, Omori Y, Iwamoto Y. Genetic polymorphism of renin-angiotensin system is not associated with diabetic vascular complications in Japanese subjects with long-term insulin dependent diabetes mellitus. Diabetes research and clinical practice. 1999; 45:41-9.

27. Wang J, Zhu X, Yang L, Liu Y, Zhou W, Li H. Relationship between angiotensinogen gene M235T variant with diabetic nephropathy in Chinese NIDDM. Chinese medical journal. 1999; 112:797-800.

28. Freire MB, Ji L, Onuma T, Orban T, Warram JH, Krolewski AS. Gender-specific association of M235T polymorphism in angiotensinogen gene and diabetic nephropathy in NIDDM. Hypertension. 1998; 31:896-9.

29. Marre M, Jeunemaitre X, Gallois Y, Rodier M, Chatellier G, Sert C, Dusselier L, Kahal Z, Chaillous L, Halimi S, Muller A, Sackmann H, Bauduceau B, et al. Contribution of genetic polymorphism in the renin-angiotensin system to the development of renal complications in insulin-dependent diabetes: Genetique de la Nephropathie Diabetique (GENEDIAB) study group. The Journal of clinical investigation. 1997; 99:1585-95. doi: 10.1172/jci119321.

30. Pei Y, Scholey J, Thai K, Suzuki M, Cattran D. Association of angiotensinogen gene T235 variant with progression of immunoglobin A nephropathy in Caucasian patients. The Journal of clinical investigation. 1997; 100:814-20. doi: 10.1172/jci119596.

31. Ringel J, Beige J, Kunz R, Distler A, Sharma AM. Genetic variants of the renin-angiotensin system, diabetic nephropathy and hypertension. Diabetologia. 1997; 40:193-9. doi: 10.1007/s001250050662.

32. Fogarty DG, Harron JC, Hughes AE, Nevin NC, Doherty CC, Maxwell AP. A molecular variant of angiotensinogen is associated with diabetic nephropathy in IDDM. Diabetes. 1996; 45:1204-8.

33. Schmidt S, Giessel R, Bergis KH, Strojek K, Grzeszczak W, Ganten D, Ritz E. Angiotensinogen gene M235T polymorphism is not associated with diabetic nephropathy. The Diabetic Nephropathy Study Group. Nephrology, dialysis, transplantation. 1996; 11:1755-61.

34. Tarnow L, Cambien F, Rossing P, Nielsen FS, Hansen BV, Ricard S, Poirier O, Parving HH. Angiotensinogen gene polymorphisms in IDDM patients with diabetic nephropathy. Diabetes. 1996; 45:367-9.
